# Wheat Blast: A Disease Spreading by Intercontinental Jumps and Its Management Strategies

**DOI:** 10.3389/fpls.2021.710707

**Published:** 2021-07-23

**Authors:** Pawan K. Singh, Navin C. Gahtyari, Chandan Roy, Krishna K. Roy, Xinyao He, B. Tembo, Kaijie Xu, Philomin Juliana, Kai Sonder, Muhammad R. Kabir, Aakash Chawade

**Affiliations:** ^1^International Maize and Wheat Improvement Center (CIMMYT), Mexico City, Mexico; ^2^ICAR-Vivekananda Parvatiya Krishi Anusandhan Sansthan (VPKAS), Almora, India; ^3^Department of Plant Breeding and Genetics, BAC, Bihar Agricultural University, Sabour, India; ^4^Bangladesh Wheat and Maize Research Institute (BWMRI), Dinajpur, Bangladesh; ^5^Zambia Agricultural Research Institute (ZARI), Chilanga, Zambia; ^6^Institute of Cotton Research, Chinese Academy of Agricultural Sciences, Anyang, China; ^7^Department of Plant Breeding, Swedish University of Agricultural Sciences, Lomma, Sweden

**Keywords:** wheat blast, *Magnaporthe oryzae* pathotype *Triticum*, disease spread, integrated disease management, Intercontinental spread

## Abstract

Wheat blast (WB) caused by *Magnaporthe oryzae* pathotype *Triticum* (MoT) is an important fungal disease in tropical and subtropical wheat production regions. The disease was initially identified in Brazil in 1985, and it subsequently spread to some major wheat-producing areas of the country as well as several South American countries such as Bolivia, Paraguay, and Argentina. In recent years, WB has been introduced to Bangladesh and Zambia *via* international wheat trade, threatening wheat production in South Asia and Southern Africa with the possible further spreading in these two continents. Resistance source is mostly limited to 2NS carriers, which are being eroded by newly emerged MoT isolates, demonstrating an urgent need for identification and utilization of non-2NS resistance sources. Fungicides are also being heavily relied on to manage WB that resulted in increasing fungal resistance, which should be addressed by utilization of new fungicides or rotating different fungicides. Additionally, quarantine measures, cultural practices, non-fungicidal chemical treatment, disease forecasting, biocontrol etc., are also effective components of integrated WB management, which could be used in combination with varietal resistance and fungicides to obtain reasonable management of this disease.

## Introduction

Rice blast is one of the most widely occurring and large-scale devastating crop diseases, with its causal pathogen *Magnaporthe oryzae* pathotype *Oryza* (MoO) ranked the first place of the 10 most devastating fungal plant pathogens (Dean et al., 2012). In comparison, wheat blast (WB) is much less known, having been confined to South America for three decades before its recent outbreak in Bangladesh (Ceresini et al., 2018). Both rice and wheat blast are caused by *M. oryzae* and are initially assumed to have the same pathogen, which is later proved to be wrong. WB is caused by *M. oryzae* pathotype *Triticum* (MoT), which is genetically different from MoO, although the two pathotypes have identical morphological traits (Cruz and Valent, 2017). Because of its limited epidemic regions, WB has been much less investigated compared with rice blast in all aspects of research. Researchers had warned of the possible expansion of the disease to other continents (Duveiller et al., 2011), and, subsequently, it was reported in Bangladesh in Asia and Zambia in Africa (Malaker et al., 2016; Tembo et al., 2020). Since then, WB has drawn increasing attention, considering its potentiality of further spreading to neighboring countries, namely, India, Pakistan, and China, which are all major wheat producers and where wheat is used as one of the major staple food crops for billions of inhabitants. Molecular analyses with MoT-specific marker and comparative genome sequencing confirmed that Bangladesh MoT isolates have a high genetic similarity to those from South America (Islam et al., 2016; Malaker et al., 2016). WB is known to have devastating effects on yield losses of up to 100% (Duveiller et al., 2016a; Cruz and Valent, 2017). Therefore, an effort is needed to stop the spread of MoT to other parts of the world because inaction may lead to a catastrophe. Active research and breeding study on WB have been conducted in the last few years, and numerous research articles have been published on every aspect of WB research, along with the release of many WB-resistant varieties in the WB-affected or threatened countries. In this review article, we have summarized the research and breeding progress for WB resistance in the last decades and suggested few future study areas, considering rapidly advancing technologies.

## Symptoms and Diagnosis of Wheat Blast

**FIGURE 1:**
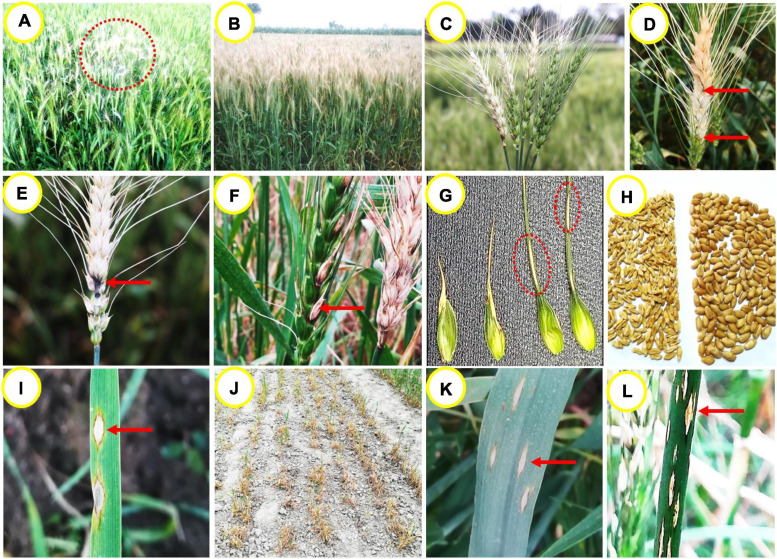
Wheat blast symptoms on different parts of the plant. **(A)** initial symptoms of blast in wheat field in a patch, **(B)** infected field showing silvery bleached spikes with green canopy, **(C)** typical partial or full bleached spikes in field, **(D)** a partially bleached spike with multiple points of infection, **(E)** dark-gray sporulation of the fungus MoT on the rachis, **(F)** infected awns show brown to whitish discoloration, **(G)** infected glumes show elliptical lesions with white to brown center and dark gray margins, **(H)** severely shriveled or wrinkled blast affected vs. healthy grains of wheat, **(I)** typical eye-shaped lesions with gray or whitish centers surrounded by dark brown margins on seedling leaf, **(J)** a severely damaged seedling field affected by MoT infection, **(K)** typical eye-shaped or elliptical lesions on a mature leaf, and **(L)** elliptical or elongated lesions on blast-affected stem having white centers surrounded by brown or blackish margins.

Initial identifiable symptom of the disease is observed at the reproductive stage of the crop in a scattered patch in wheat field (Figure 1A). With time, the patches coalesce and the whole field is severely damaged. Spikes in the infected field become silvery color while the leaves may remain green (Figure 1B; Singh, 2017). The fungus MoT can infect all above-ground parts of wheat such as spike, leaf, peduncle, glume, awn, and seed (Igarashi, 1990; Urashima et al., 2009; Cruz et al., 2015; Cruz and Valent, 2017), but the most distinguishable symptom is observed on the spikes (Malaker et al., 2016; Saharan et al., 2016; Cruz and Valent, 2017). Partial or complete bleached spikes are the most notable symptoms of wheat blast, starting from an apparent blackish-gray-colored infection point at rachis or the base of infected spikes (Figure 1C). Depending on the place of infection on the spike, partial or full drying takes place. Sometimes, multiple points of infection in a single rachis can be observed under high inoculum pressure in susceptible cultivars (Figure 1D). An infection in the rachis or peduncle can block the nutrient transportation system of the plant and ultimately damage all the upper spikelets above the infection points (Cruz and Valent, 2017). At the point of infection of the rachis, gray or dark-gray or black sporulation of the fungus can be observed in highly susceptible cultivars (Figure 1E; Igarashi, 1990; Islam et al., 2016). Infected awns show brown to white stain, while glumes show elongated lesions with reddish brown to dark gray margins and white to light brown center (Figures 1F,G; Saharan et al., 2016; Cruz and Valent, 2017). During sporulation, lesions have gray centers that become white to tan after the release of spores (Igarashi et al., 1986; Igarashi, 1990). The extent of wheat blast damage on grains depends upon the timing and intensity of the infection. Infection occurring prior to anthesis or at an early stage of flowering results in total sterility of spikes, thereby resulting in seed abortion (Goulart et al., 1990; Goulart and Paiva, 1992; Urashima et al., 2009). Infection at the grain filling stage results in small, wrinkled, deformed, and low test weight kernels (Figure 1H; Goulart et al., 2007; Malaker et al., 2016), which become unfit for human consumption (Urashima et al., 2009).

Under field conditions, lesions on the leaves may vary in shape and size depending on the crop growth stage. Leaves of highly susceptible cultivars can be infected severely at the seedling stage and lead to total plant death under conducive weather conditions (Igarashi, 1990; Singh, 2017). Resistant cultivars may also show moderately susceptible to susceptible reaction to the disease at the seedling stage (Roy et al., unpublished). First visible symptom on young seedling includes water-soaked diamond shaped lesion which turns grayish white center with dark brown border with disease progression (Figure 1I). When several lesions coalesce, the entire leaf could die (Figure 1J; Rios et al., 2013). The old leaves are more susceptible to MoT than the young ones (Cruz et al., 2015), in conducive environments in highly susceptible cultivars. Symptoms on the leaf include the presence of elliptical or elongated or eye-shaped, grayish to tan necrotic lesions with dark borders (Figure 1K; Malaker et al., 2016). Lesions can also be rarely seen on the leaf collar, culm, culm node, and stem. Stem lesions include those that are elongated or elliptical in shape with a white center surrounded by a dark-brown or blackish margin (Figure 1L).

**FIGURE 2:**
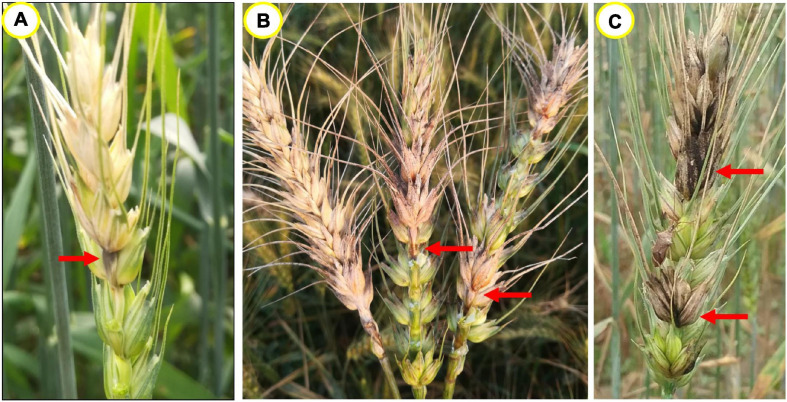
Blast and blast-like symptoms on wheat heads. **(A)** A typical blasted head having gray colored infection point, **(B)** symptoms of FHB showing superficial pink to orange masses of spores of *F. graminearum* with pink colored infection points, and **(C)** symptoms of spot blotch giving black discoloration on the infected spikelets because of *B. sorokiniana*.

Wheat head blast in the field sometimes can be wrongly diagnosed, because it somewhat resembles Fusarium head blight (FHB) and spot blotch, caused by *Fusarium graminearum* and *Bipolaris sorokiniana*, respectively (Pieck et al., 2017; Singh, 2017). When the rachis is infected with FHB, spikelets above the infection point may also become bleached, with pink to orange masses of spores of the fungus, in contrast to the gray masses of MoT (Figure 2A), being observed on the infected spikelets (Figure 2B; Wise and Woloshuk, 2010; Valent et al., 2016). In the case of spot blotch, dark brown or black discoloration develops on the infected spikelets and such spikes may possess healthy spikelets at both ends from the infection point (Figure 2C). In the field, blast symptoms on the leaves are often unidentifiable because of the mixed infection of spot blotch.

Traditional disease diagnosis based on pathogen morphology is not reliable, since MoT cannot be morphologically distinguished from other *M. oryzae* pathotypes (Thierry et al., 2019, 2020). Therefore, molecular diagnosis of MoT is of utmost importance for disease diagnosis and subsequent management. Pieck et al. (2017) have reported a polymerase chain reaction (PCR) assay based on MoT3 primer sets, and Yasuhara-Bell et al. (2018) converted it to a loop-mediated isothermal amplification (LAMP) assay, enabling rapid detection of MoT in both laboratory and field conditions. The MoT3 marker was recently used to reveal that MoT causes blast on some other hosts such as triticale (Roy et al., 2020b), barley (Roy et al., 2021a), and durum (Roy et al., 2021b). However, this marker could produce false negative results in MoT isolates lacking the MoT3 locus, e.g., BR0032. To address this problem, Thierry et al. (2019, 2020) have developed a tool kit with novel markers for ordinary PCR, qPCR, and LAMP that have shown good specificity to MoT, although false positive results were observed in few non-MoT isolates. Based on genome sequence comparison of MoO and MoT, Kang et al. (2020) identified two DNA fragments specific to MoT and developed markers for a set of rapid diagnostic tools, which, unfortunately, also exhibited false positive results. Therefore, no perfect diagnostic tool is currently available for MoT, and it is beneficial to apply multiple markers for cross validation.

## Production Losses

Wheat blast is one of the most devastating and yield limiting disease in warm and humid wheat production regions. The economic importance of this disease arises because it reduces grain yield and quality drastically (Goulart et al., 2007). The maximum yield damage happens when spike infection occurs during anthesis or early grain filling stage (Goulart et al., 2007) and/or when the fungus attacks at the base of the spike, thereby restricting the development of the grains and killing the head completely (Kohli et al., 2011). Yield losses can reach up to 100% when a susceptible cultivar is grown under late sown conditions in Bangladesh and under early sown conditions in South America (Roy et al., unpublished; He et al., 2020b). The losses due to the disease depend upon several factors such as genotype, crop growth stage, planting date, weather conditions (temperature, humidity, rainfall, etc.), and disease severity (CIMMYT, 2016; Cruz and Valent, 2017).

In South America, the losses in grain yield were estimated in the range of 10–100% (Duveiller et al., 2016a). In 1987, yield losses incurred in three Brazilian states (Parana, Matto Grosso do Sul, and Sao Paulo) varied between 10.5 and 53% (Goulart and Paiva, 1992), thereby influencing farmers not to grow wheat (Callaway, 2016). The first outbreak of WB in Bolivia in 1996 resulted in almost 80% of production loss (Barea and Toledo, 1996). In the subsequent year (1997), the disease again devastated the early planted crops causing 100% yield loss, which was responsible for the sharp decline in wheat area production in subsequent years in Bolivia. In Paraguay where the first epidemic occurred in 2002, production losses of more than 70% were recorded in the early broadcasted fields (Viedma and Morel, 2002). Most of the harvested grain did not meet marketable values for test weight and had to be used as animal feed. In 2016 in Bangladesh, the overall yield loss estimates by the Department of Agricultural Extension were close to 50% in about 15,000 ha affected, which posed a significant threat to the aggregate wheat production of the country (Islam et al., 2016). The disease reappeared in the subsequent years (2017–2020) with comparatively lower disease severity, and an insignificant yield loss (1–5%) was incurred because of unfavorable weather conditions and the adoption of different management packages.

## Pathogen Biology

The causal organism of wheat blast is a haploid, filamentous, ascomycetous fungus named *Magnaporthe oryzae* B.C. Couch and L.M. Kohn (anamorph *Pyricularia oryzae* Cavara) (Couch and Kohn, 2002). Because of its self-incompatibility, the fungus reproduces sexually only when there is crossing between two sexually compatible and fertile individuals (Maciel et al., 2014; Maciel, 2019). This happens once the female receptive structure termed ascogonium is able to accept the compatible nucleus or nuclei of the male benefactor *via* conidia or receptor hyphae (Kang et al., 1994; Moreira et al., 2015). The fungus is very much host-specific and cannot infect incompatible hosts. Based on host specificity, mating type, and genetic similarity, isolates of *M. oryzae* are subdivided into several pathotypes (Urashima et al., 1993; Kato et al., 2000; Tosa et al., 2004; Tosa and Chuma, 2014). Among the pathotypes, *Oryza* is responsible for infecting rice, *Setaria* for foxtail millet, *Eleusine* for finger millet, *Panicum* for proso millet, *Triticum* for wheat, *Avena* for oat, *Lolium* for perennial and annual ryegrass, and many other ones for grasses (Kato et al., 2000; Farman, 2002; Tosa et al., 2004; Maciel, 2019). It has been proved that MoT is distinct from other host-specific pathotypes based on host range (Prabhu et al., 1992; Urashima et al., 1993), sexual fertility (Urashima et al., 1993), and DNA fingerprinting (Urashima et al., 1999; Urashima et al., 2005). Isolates from each host are entirely pathogenic on their original host genus (Tosa et al., 2006). The aforementioned pathotypes are genetically close and interfertile and were distinct from the *Digitaria* isolates originally designated *P. grisea* (Urashima et al., 1993; Kato et al., 2000; Murakami et al., 2000; Tosa et al., 2004, 2006), which was later confirmed with a multilocus phylogenetic analysis (Kato et al., 2000; Couch and Kohn, 2002). It is noteworthy that MoT attacks not only wheat but also its relative triticale, barley, and durum (Roy et al., 2020b, 2021a, b). There is no cross infection that happened between rice and wheat blast isolates on either of the alternative host (Prabhu et al., 1992; Tosa et al., 2004). The *Triticum* pathotype population evolves fast, resulting in a level of genetic diversity that is higher than that of other pathotypes (Urashima et al., 2005; Tosa et al., 2006; Maciel et al., 2014; CIMMYT, 2016).

**FIGURE 3:**
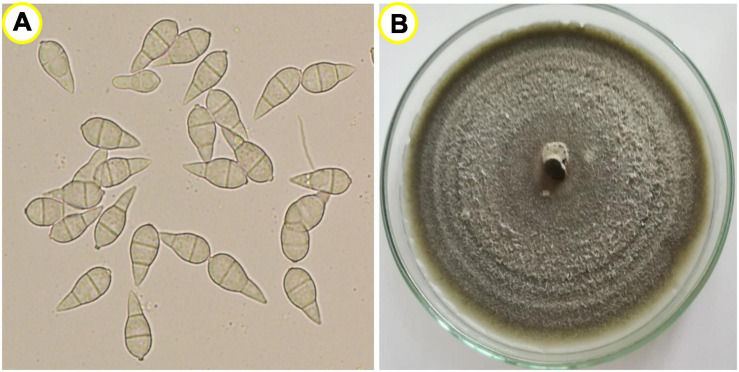
**(A)** Pyriform two-septate hyaline to pale, gray-colored asexual conidia under compound microscope (magnification 400×) and **(B)** dark gray-colored colony of the fungus grown on potato dextrose agar (PDA) medium.

The fungus produces pear-shaped two-septate three-celled asexual conidia, which are hyaline to pale gray-colored (Figure 3A). The conidia are produced in clusters on long septate, slender conidiophores in a sympodial manner. Conidiophores are light brown in color, solitary, and erect. Mycelia are thin, slightly brownish, septate, and highly branched. The fungus can be purified by isolation of a single conidium; and when grown in pure culture, the fungal colony appears white, light gray, or dark gray (Figure 3B). During infection, the conidia of the fungus are attached to the plant surface by producing a polarized germ tube and then start to germinate on the leaf surface by 6 h of attachment from both apical and basal cells, followed by swelling at the tip of germ tube known as appressorium, which helps to penetrate into the leaf epidermis or rachis cuticle, and then followed by further invasive hyphal expansion to colonize plant tissues (Tufan et al., 2009). The fungus also secrets antibiotics and mycotoxin, which help to colonize in plant tissue for successful biotrophic growth (Patkar et al., 2015; Yan and Talbot, 2016). Sexually fertile *M. oryzae* strains also produce small, crescent-shaped microconidia, which are produced from phialides, but their role for plant infection in nature is largely unknown (Zhang et al., 2014).

## Spread of Wheat Blast in South America

**FIGURE 4:**
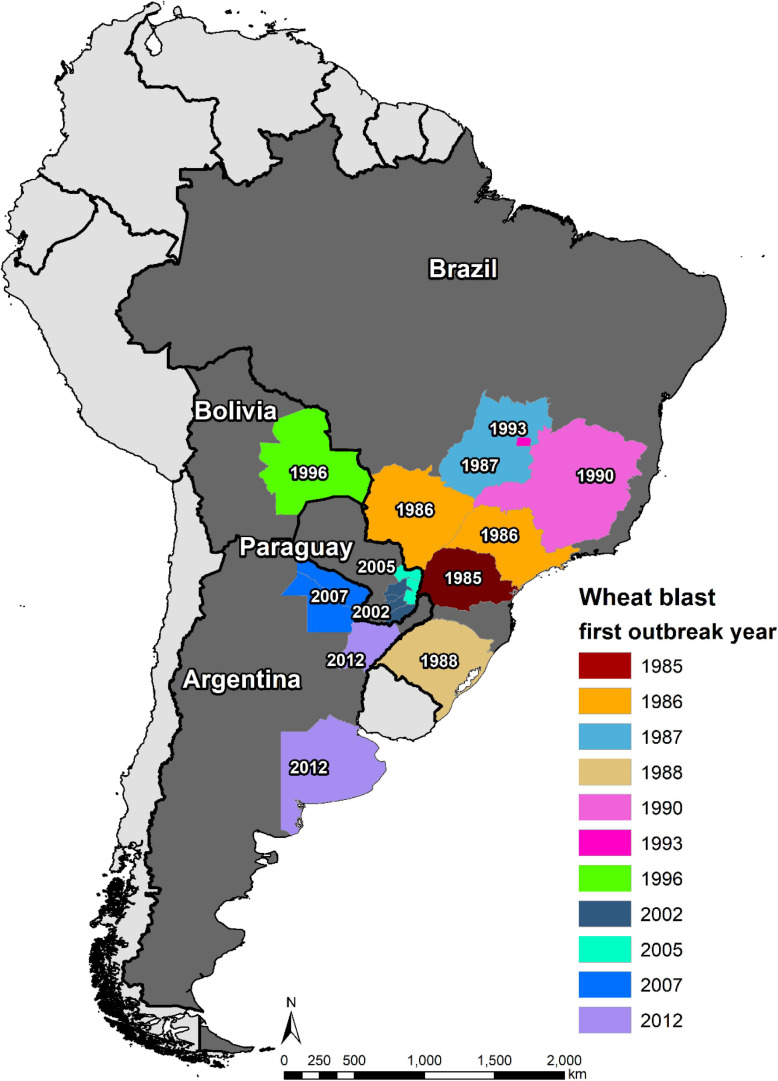
Spread of wheat blast in South America from 1985 to 2021.

The first WB epidemic occurred in 1985 in the state of Paraná, one of major wheat producer of Brazil, affecting its six northern municipalities, i.e., Primeiro de Maio, Sertanópolis, Rancho Alegre, Londrina, Engenheiro Beltrão, and São Pedro do Ivaí (Igarashi et al., 1986). In 1986, WB spread northward from Paraná to its neighbor states São Paulo and Mato Grosso do Sul, resulting in 27 municipalities in the three states being affected by the disease (Picinini and Fernandes, 1990). By 1987, WB was present in more than 70 municipalities in Paraná, causing a yield loss of 10–12% (Goulart et al., 1990). In the same year, it spread further northward to the state of Goias, where the disease was observed in Vicentinopolis (Prabhu et al., 1992). In order to have better knowledge of the epidemic region, an intensive field survey was conducted in Mato Grosso do Sul in 1988, and the results indicated an occurrence in 14 municipalities, namely, Dourados, Ponta Porã, Rio Brilhante, Itaporã, Fátima do Sul, Douradina, Maracaju, Caarapó, Aral Moreira, Bonito, Nova Andradina, Naviraí, Amambai, and Sidrolândia (Goulart et al., 1990). Being a neighbor to three WB affected states, the state of Minas Gerais was soon declared to be also affected by the disease in 1990 (Ceresini et al., 2018). Then, the incidence in 1993 in the capital city Brazilia, located between the states of Goias and Minas Gerais, was not surprising (Dos Anjos et al., 1996). Southward, WB arrived in 1988 in the other major wheat producer area, the state of Rio Grande do Sul, and was first found in northern municipality Lagoa Vermelha (Picinini and Fernandes, 1990). Nowadays, the disease is present in all wheat production zones in Brazil (Figure 4) because of both natural spread and lack of strict seed quarantine measures among states; and the latter served as the main cause of the WB outbreak in Paraguay, Bolivia, and Bangladesh (Ceresini et al., 2018).

The main wheat production zones of Paraguay lay in the east and south, which border to two main WB epidemic states of Brazil, Paraná and Mato Grosso do Sul. Therefore, WB could have easily spread from Brazil to Paraguay even if there was no introduction of MoT-contaminated seeds. Indeed, the first incidence of the disease was observed in the border region of the two countries in 1987, only 2 years after the appearance in Brazil (Cunfer et al., 1993). However, the first WB epidemic and official report occurred in 2002, causing yield losses of up to 80% in early sown fields, with the Itapúa Department being the most severely affected, followed by the Alto Paraná Department (Viedma, 2005). In 2005, another severe epidemic of WB hit Paraguay, affecting about 10,000 ha in Alto Paraná, Canindeyú, etc. (Viedma et al., 2010). Currently, the WB-affected regions in Paraguay include Alto Paraná, Itapúa, Caaguazú, Caazapá, Canindeyú, and Guairá Departments (Ceresini et al., 2018), covering most of the wheat production zones in the country.

The first WB epidemic in Bolivia was recorded in 1996 in the lowland Santa Cruz region, resulting in about 80% of yield reduction (Barea and Toledo, 1996). The disease was more devastating in 1997, causing 100% yield loss in early sown fields and substantial decline in the wheat area in Santa Cruz in subsequent years (Kohli et al., 2011). So far, WB has occurred mostly in the lowland wheat fields in Santa Cruz, which is the most important wheat producer in Bolivia, whereas those in highlands are not severely affected (Vales et al., 2018).

The first WB incidence in Argentina was in its north-eastern province Formosa in 2007, followed by reports from two other northeastern provinces, Chaco (2007/08) and Corrientes (2012/13), all bordering to Paraguay (Kohli et al., 2011; Gutiérrez and Cúndom, 2015). The occurrence of this disease in the above-mentioned three provinces did not pose a big threat to Argentine wheat production because of the limited wheat area there; however, the disease arrived in the province of Buenos Aires in 2012, one of its major wheat producers (Perelló et al., 2015). This ignited a series of research activities on the disease in Argentina, although large-scale yield loss due to WB has not been reported yet (Perelló et al., 2017).

## Spread of Wheat Blast in Bangladesh, South Asia

**FIGURE 5:**
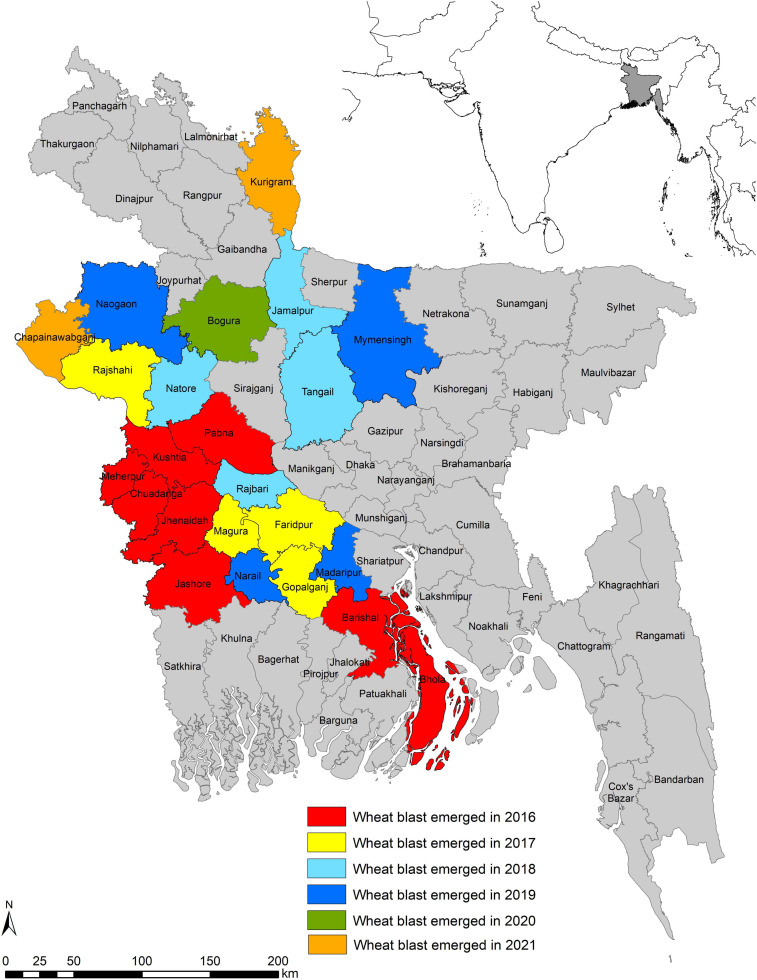
Spread of wheat blast in Bangladesh from 2016 to 2021.

The incidence of WB in February 2016 came as a sudden shock, taking the South Asia wheat production regions off-guard when a series of reports (Callaway, 2016; Islam et al., 2016; Malaker et al., 2016) confirmed the epidemic presence in eight districts, namely, Barishal, Bhola, Chuadanga, Jashore, Jhenaidah, Kushtia, Meherpur, and Pabna in the southwestern and southern districts of Bangladesh. This first incidence beyond South America affected nearly 15,000 ha (3.5% of total 0.43 million ha wheat area in Bangladesh) with an average yield loss of 25–30% (Islam et al., 2016; Malaker et al., 2016). In the subsequent 5 years (2017–2021), weather conditions during the wheat croppingseason were cooler and drier, and did not favor WB infection, development, and spread (Mottaleb et al., 2019; BWMRI, unpublished). Still, the disease did not remain confined to the initial eight affected districts but spread further to 14 new districts (Figure 5). In 2017, it spread to four new districts, Rajshahi, Faridpur, Magura, and Gopalganj, which are adjoined to previously WB-affected districts. In 2018, the disease spread to four more new districts: Tangail, Jamalpur, Natore, and Rajbari. Among these districts, Jamalpur is not adjacent to any of the previously affected ones. In 2019, there was a further spread of WB to Naogaon, Mymensingh, Madaripur, and Narail districts. In 2020, a new district, Bogura, which is close to the northwestern part of the country and is considered as the major wheat-producing region of the country (BWMRI, 2020), was reported to have WB. In 2021, the disease has further spread to two districts, Kurigram and Chapainawabganj, but the infection levels are very low. The pattern of disease expansion clearly indicated that both the seed-borne and air-borne means of dispersal is happening in Bangladesh. Mottaleb et al. (2018) identified several warmer and humid districts in Bangladesh as vulnerable to WB. In the last few years, it has been observed that seven districts, namely, Tangail, Jamalpur, Naogaon, Mymensingh, Kurigram, Chapainawabganj, and Bogura, which are located in the northern part of Bangladesh where relatively cooler conditions prevail, were not predicted as vulnerable to WB, but incidence of the disease was observed in these districts. This scary situation of WB being identified in cooler and drier conditions enhanced the vulnerability of South Asia to WB and indicated the ability of MoT to survive under harsh conditions.

## Spread of Wheat Blast in Zambia, Africa

**FIGURE 6:**
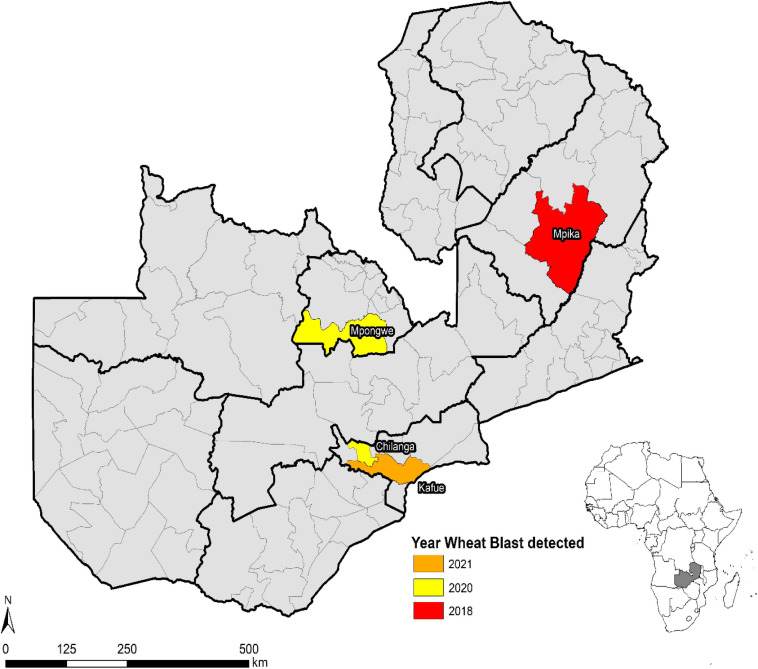
Spread of wheat blast in Zambia from 2018 to 2021.

Wheat blast was first observed in Zambia in February 2018 during the rainfed season in Mpika district of Muchinga province (Figure 6). During the 2017–2018 season, disease incidence and severity were high because of favorable weather conditions supporting the disease development and pathogen proliferation. However, low disease severity was observed during the2018–2019 season in experimental and farmer fields in Mipika district because of hot and dry conditions. During the 2019–2020 crop season, high disease incidence and severity were observed in both experimental (Mt. Makulu, Mpika, and Mpongwe) and farmer fields (Mpika). In some areas where the climatic conditions were hot and dry, disease incidence and severity were low. In the 2020–2021 crop season, the disease was observed in experimental fields (Mt. Makulu, Mpika, and Mpongwe) and also at a farmer field in Kafue district that grew the susceptible variety Coucal. The spread of WB in Zambia could be ascribed more as seed-borne rather than air-borne spread.

## Areas Vulnerable to Wheat Blast Across the World

**FIGURE 7:**
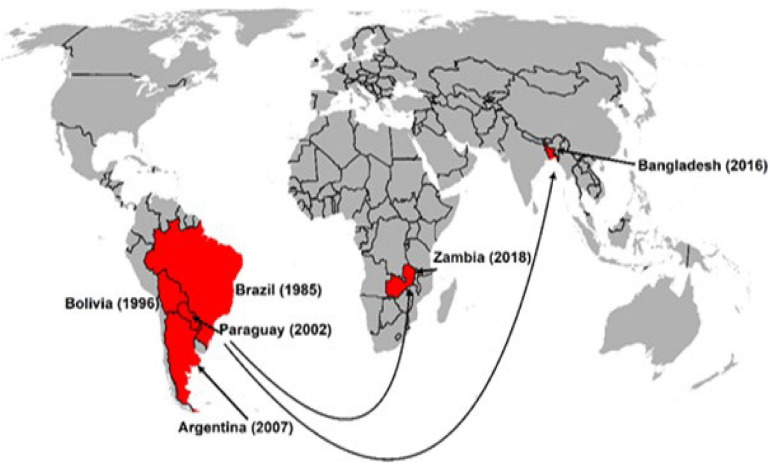
Intercontinental spread of wheat blast attributed to grain trade.

First, Duveiller et al. (2011) estimated the risk of wheat blast in other continents and observed areas of high risk in parts of Central India, Bangladesh, Ethiopia, Eurasia, and North America using a climate similarity approach. Cao et al. (2011) predicted vulnerable regions in mid-east South America, southeast and midwest Africa, southeast of South Asia, east coast of Australia and south China, using the MaxEnt model. Kohli et al. (2011) forecasted that climatic changes associated with global warming could make WB spread to other parts of the world, and that WB invasion of the Asian continent is likely to cause devastating effects unless immediate control measures are taken. Unfortunately, their predictions came true in 2016 with the WB outbreak in Bangladesh. Since 2016, several studies on the vulnerability of wheat growing areas to wheat blast have been published. Studies using different models have revealed the vulnerable areas in Bangladesh, India, China, and Pakistan (Sadat and Choi, 2017; Mottaleb et al., 2018). In another study, Duveiller et al. (2016b) cited that further spread of WB in Latin America is possible with vulnerable areas in Mexico, Ecuador, and Andean valleys. Cruz et al. (2016) observed that several southeastern states (Louisiana, Mississippi, and Florida) in the United States are vulnerable to WB. Factors such as global warming, irregular rains, cultivation of susceptible cultivars and unrestricted wheat grain movement especially from countries with cases of WB, the increasing virulence of the pathogen, and its fungicide resistance, potential sexual recombination, and possible cross-host infections could lead to more frequent outbreaks and spread of the disease to other major wheat-producing countries. Grain trade has been attributed to the spread of WB from South America to Bangladesh and Zambia (Figure 7). Ceresini et al. (2018) further alerted that strengthening quarantine and biosafety regulations to prevent further spread in Asia or introduction of WB into other wheat-growing regions of the world, such as Europe, Australia, and North America, should be of the highest priority.

## Management Strategies

Wheat blast is a very challenging disease to manage, and no single strategy is capable of achieving a satisfactory level of management. Therefore, for blast-free areas, quarantine measures are paramount to preclude the introduction of the disease, which would otherwise be impossible to eradicate. Areas adjoining endemic regions may adopt a wheat holiday concept to limit the disease spread. For endemic WB regions, an integrated management approach is recommended, such as varietal resistance, fungicide application, cultural management, non-fungicidal chemical treatment, and biocontrol methods. These strategies are described in the sections below.

## Containment and Quarantine

Quarantine is one of the best approaches to restrict the spread of a pathogen from endemic regions to disease-free areas/countries and to avoid potential outbreaks in new regions. Aerial dispersal of the disease is limited as heavier asexual spores are reported to travel up to 1 km (Urashima et al., 2007), whereas sexual spores are lighter and, hence, may be able to travel much longer distances (Maciel et al., 2014). Thus, infected seeds are the most probable source for disease introduction and spread through large intercontinental distances. Failure of proper quarantine and seed trade laws led to the WB pathogen into Bangladesh (Islam et al., 2016). This strongly implies a likewise invasion of the pathogen to WB-prone regions in South Asian countries such as India and Pakistan. Under an assumption of favorable climatic conditions, Mottaleb et al. (2018) predicted a vulnerability of 17% of cultivated wheat area or 0.88 million tons of yield loss in South Asia. An early study exhibited that the MoT pathogen can survive in seeds for up to 22 months (Reis et al., 1995). Hence, it is imperative for nations vulnerable to the disease to meticulously draft and execute their seed entry and quarantine laws. Seeds from endemic areas may be prohibited for entry. Also within an endemic region, laws can also be framed so that locally produced wheat seeds are not used as seed and do not go to flour industries for direct consumption.

## Wheat Holiday

Wheat holiday is the suspension of wheat cultivation in blast-affected areas or vulnerable areas with a high probability of disease dispersal. It encompasses the forceful ban of wheat cultivation by the respective government with an intention to stop the spread of the disease to adjoining areas. However, most farmers in WB-affected areas in Bangladesh are small and resource-poor, and, hence, there is a need to give alternative cropping plans to them to make a wheat holiday successful in holistic terms. India has banned wheat cultivation within 5 km from the Bangladesh boundary and instead directed for growing of legumes and oilseeds crops. The state government of West Bengal (Indian state adjoined to Bangladesh) has prohibited wheat cultivation in two districts (Murshidabad and Nadia) for 3 years (ICAR-IIWBR, 2020). Similarly, in Bangladesh, a study by Mottaleb et al. (2019) indicated the feasibility of maize, onion, garlic, and lentil as profitable alternative crops to wheat. While considering any alternative plan, it is important that the crops substituted should not act as an alternative to MoT. Also, for a successful “wheat holiday” management strategy, the alternative hosts of MoT such as weeds should be controlled or avoided, which is very challenging.

## Breeding for Resistance

### Understanding the Enemy (Pathogen)

While breeding for blast resistance in wheat, it is important to understand pathogen diversity, host specificity, and evolution. The fungus *M. oryzae* is reported to infect 137 species in the Poaceae family (Choi et al., 2013). Various pathotypes are named after the host crop species infected by the pathogen such as *Oryza*, *Eleusine*, *Avena*, and *Lolium*. Apart from crop species, it infects various weeds and grasses such as *Cenchrus echinatus*, *Digitaria sanguinalis*, and *Echinochloa crus-galli* (Kohli et al., 2011). Compared with *M. oryzae* pathotype *Oryza*, sexual reproduction is more frequent in MoT, as reflected in its high diversity found in fields (Urashima et al., 1999). However, the predominance of only one mating type suggests asexual reproduction as the predominant mode of reproduction (Urashima et al., 2017). Maciel et al. (2014) reported a mixed reproductive system occurring for MoT where a best-fit strain produced by sexual reproduction is maintained generation after generation by asexual reproduction. Strains collected between 2016 and 2017 in Bangladesh indicated a single genotype, implying its asexual propagation in the natural environment, which is in sharp contrast to the situation in South America where high genetic diversity was found among MoT isolates (Ceresini et al., 2018).

### Sources of Host Resistance

Ever since the first wheat blast outbreak in Brazil in 1985, efforts on identification of resistance sources in both common wheat and its relatives have been exerted. Several promising varieties were identified in early studies, but they all became susceptible in later experiments (Igarashi, 1990; Urashima and Kato, 1994). A subsequent screening study in Brazil led to the identification of few moderately resistant varieties such as BRS 49, BRS 120, BRS 220, and IAPAR 53 (Prestes et al., 2007). In Bolivia and Paraguay, identification of resistant varieties relied mostly on field observation over years, from which several moderately resistant varieties have been identified, e.g., Sausal CIAT, Motacu CIAT, Patuju CIAT, and Urubo CIAT in Bolivia, and Caninde 1 and Itapua 75 in Paraguay (Buerstmayr et al., 2017). It was found that many such resistant varieties have the CIMMYT genotype Milan in their pedigree, and later research indicated that 2NS translocation was the underlying resistance factor (Cruz et al., 2016), which was initially introduced from *Ae. ventricosa* to a French variety “VPM1” (Helguera et al., 2003). This translocation is frequent in the CIMMYT germplasm as it confers a wide range of resistance against stripe rust (*Yr17*), leaf rust (*Lr37*), stem rust (*Sr38*), cereal cyst nematode (*Cre5*), root-and knot nematodes (*Rkn3*), and also has increased yield potential (Cruz et al., 2016; Juliana et al., 2020). However, the translocation exhibited different phenotypic effects across wheat lines, signifying the role of genetic background in its expression (Cruz et al., 2016; He et al., 2020a, 2021). “BARI Gom33,” a zinc bio-fortified blast-resistant wheat variety released in Bangladesh in 2017 is a success story of utilizing the 2AS/2NS translocation in breeding (Hossain et al., 2019). However, too much dependency on this translocation in the form of large acreages in South Asia and South America is making it vulnerable against new MoT isolates because of strong directional selection. Virulent strains on this translocation have been reported across South America (Cruz et al., 2016), such as the highly virulent Brazilian strain 16MoT001 reported by Cruppe et al. (2020), making it imperative to look out for novel resistance sources.

Non-2NS resistance sources having moderate levels of resistance have been identified in both field and controlled conditions (He et al., 2021). Few accessions of wild relatives of wheat, namely, *Ae. tauschii* and *Ae. umbellulata*, were found to have resistance against WB (Buerstmayr et al., 2017). Resistant (TA10142) and moderately resistant (TA-1624, TA-1667, TA10140) *Ae. tauschii* accessions were identified using field and greenhouse phenotyping facilities in Bolivia, Brazil, and the United States (Cruppe et al., 2020). Identification of resistance in wild relatives indicates the potential use of synthetic hexaploidy wheat (SHW) varieties against the WB disease. CIMMYT SHW derivatives Patuju CIAT and Motacu CIAT were released in Bolivia because of their blast resistance (Buerstmayr et al., 2017). Many of the identified resistant sources need to be widely tested considering the influence of environmental conditions, e.g., BR 18-Terena and BRS229 are non-2NS wheat varieties widely used as parents in Brazil owing to their high head blast resistance (Ferreira et al., 2020), but they exhibited susceptibility in some environments (Ceresini et al., 2019). While breeding for WB resistance, consideration of farmers must also be accounted. A good example of it is the wide cultivation of the “Motacu” variety in Bolivia. Though this variety is moderately susceptible, it is still liked by farmers because of its earliness (Vales et al., 2018).

Considering insufficient WB resistance of the non-2NS sources, currently, it is advised to utilize such resistance together with 2NS to achieve a satisfactory WB resistance. With the identification of more non-2NS resistance, such sources could be crossed with each other to accumulate minor non-2NS resistance genes to achieve high and durable WB resistance, just as recommended in breeding for durable rust resistance (Singh et al., 2016). By that time, 2NS could be used at a lower frequency to reduce its directional selection on 2NS-virulent MoT isolates, so that its resistance could remain effective for a longer time. Breeding for seedling resistance is a target that has not received sufficient attention, mainly because of less conducive environmental conditions during the seedling stage in WB-affected regions. However, with the changing climate, the situation may change in the future, bringing new challenges in WB epidemic regions. It has been well demonstrated that WB resistance at the seedling stage does not correspond well with that at the adult plant stage (Cruz et al., 2012), emphasizing the necessity of conducting WB evaluation at both stages. The advantage of seedling evaluation is that it can be conducted in a greenhouse with high throughput and is, thus, less expensive compared with field screening. A good strategy may be to select only lines with seedling resistance for field evaluation, which could significantly reduce the workload for field trials. However, this may neglect lines with poor seedling resistance but good adult plant resistance; therefore, for regions without major issues on seedling blast, field evaluation on head blast should still be preferred.

International collaboration is much needed for screening and identifying novel sources of resistance. The formation of WB consortium in the year 2010 and databases such as the OpenWheatBlast project were a step forward in the direction. Likewise, institutes in Bangladesh in collaborations with CIMMYT, Swedish University of Agricultural Sciences, National Research Council Canada, The Sainsbury Laboratory, UK, and the University of Exeter are working on novel genome editing technologies for WB resistance (Singh *personal communication*). “Precision Phenotyping Platforms (PPP)” are established in Bangladesh and Bolivia with the help of CIMMYT and NARS partners to evaluate germplasm from across the globe in search of novel blast resistance materials, especially those of non-2NS. The government of India is utilizing PPPs and identified five resistant varieties, namely, HD3249, HD3171, HD 2967, DBW 252, and DBW 187, which have been recommended to farmers of disease-prone areas in West Bengal adjoined to Bangladesh (ICAR-IIWBR, 2020). Bangladesh released two blast-resistant varieties, BARI Gom 33 and WMRI Gom 3, Nepal released Borlaug 2020, and Bolivia released INIAF Tropical and INIAF Okinawa as blast-resistant varieties within the last 5 years.

### Resistance Mechanism: Major vs. Minor Genes

Understanding and identification of “R” (resistance) genes in host and avirulence/virulence genes in MoT is the cornerstone for successful breeding. Effectors coded by avirulence (AVR) genes are recognized by the “R” gene products of the host plant to confer resistance. The identified AVR and R genes in the WB pathosystem are very limited compared with those in rice blast. Comparative transcriptomics studies can help in hunting for new AVR and R genes effective in WB (Ferreira et al., 2020). Genetic studies have pointed toward the important role of AVR and minor pathogenicity genes in conditioning the virulence of blast pathogens, where loss of AVR and selective accumulation of minor pathogenicity genes help in slowly evolving an *M. oryzae* strain to adapt new host species (Cruz and Valent, 2017). Avirulence genes in MoO (*PWT1*, *PWT2*, and *PWT5*), MoS (*PWT1* and *PWT2*), MoA (*PWT3* and *PWT4*), and MoL (*PWT3*) confer avirulence to wheat crop (Table 1), whereas none of the MoT isolates have any of these AVR genes (Cruz and Valent, 2017).

**TABLE 1:** List of identified and postulated resistance genes in wheat against different *Magnaporthe oryzae* pathotypes.

Resistance gene/locus	Chromosome	Donor genotype	Corresponding AVR gene	Corresponding pathotype^&^	References
*Rmg1* (*Rwt4*)	1D	Norin 4	*PWT4*	MoA	Takabayashi et al., 2002
*Rmg2*	7A	Thatcher		MoT	Zhan et al., 2008
*Rmg3*	6B	Thatcher		MoT	Zhan et al., 2008
*Rmg4*	4A	Norin 4		MoD	Nga et al., 2009
*Rmg5*	6D	Red Egyptian		MoD	Nga et al., 2009
*Rmg6* (*Rwt3*)	1D	Norin 4	*PWT3*	MoL, MoE, MoA	Vy et al., 2014
*Rmg7*	2A	*T. dicoccum* lines KU112, KU120, and KU122	*AVR-Rmg8*	MoT	Tagle et al., 2015
*Rmg8*	2B	S-615	*AVR-Rmg8*	MoT	Anh et al., 2015
*RmgTd(t)*	7B	*T. dicoccum* KU109		A mutant progeny of MoA and MoT	Cumagun et al., 2014
*RmgGR119*		GR119		MoT	Wang et al., 2018
*Rwt1*^#^			*PWT1*	MoS, MoO	Tosa et al., 2006; Chuma et al., 2010
*Rwt2*^#^			*PWT2*	MoS, MoO	Murakami et al., 2000; Tosa et al., 2006
*Rwt5*^#^			*PWT5*	MoO	Tosa et al., 2006
2NS/2AS translocation	2AS/2NS	*Ae. ventricosa*		MoT	Cruz et al., 2016

*^&^MoA represents the Avena pathotype of M. oryzae, MoT the Triticum pathotype, MoD the Digitaria pathotype, MoL the Lolium pathotype, MoE the Eleusine pathotype, MoS the Setaria pathotype, and MoO the Oryza pathotype.*

*^#^These three genes have not been identified in wheat.*

The identified resistance genes can be categorized into non-host resistance genes and host resistance genes. Non-host resistance genes are the “R” genes in wheat conferring resistance against the non-MoT isolates, whereas host resistance genes are effective against MoT. Some of the identified non-host resistance genes protecting the wheat plant against non-host isolates include *Rmg1* against MoA (Takabayashi et al., 2002), *Rmg4* and *Rmg5* against *M. oryzae Digitaria* isolates (Nga et al., 2009), and *Rmg6* against MoL isolates (Vy et al., 2014). *Rmg6* was identified on chromosome 1D in wheat variety Norin4 and is effective against MoL having the *AVR* gene *PWT3* (Table 1). A host jump of an *M. oryzae* lineage to wheat was exemplified in “Anahuac,” a widespread Brazilian variety in the 1980s. This variety lacking *Rmg6* (*Rwt3*) is susceptible to MoL with *PWT3*. Therefore, MoL population massively built up on the variety and mutations occurred in *PWT3*, resulting in *pwt3-*carrying MoL isolates that are virulent even to wheat cultivars with *Rmg6*, turning MoL into MoT (Inoue et al., 2017). *RmgTd(t)* was detected by a mutant isolate from a cross between MoA and MoT, which was avirulence to most bread and durum wheat cultivars barring few susceptible tetraploid wheat cultivars (Cumagun et al., 2014).

Host resistance genes identified so far include *Rmg2*, *Rmg3*, *Rmg7*, *Rmg8*, and *RmgGR119* (Table 1). *Rmg2* and *Rmg3* located on chromosomes 7A and 6B, respectively, were found to be effective seedling resistance genes detected in the variety “Thatcher” (Zhan et al., 2008). However, they were not effective at the head stage, and their resistance had been overcome by new MoT strains (Cruppe et al., 2020). *Rmg7* was identified on chromosome 2A in tetraploid wheat (Tagle et al., 2015), whereas *Rmg8* was detected on chromosome 2B in hexaploid wheat (Anh et al., 2015). They had a common *AVR* gene, i.e., *AVR-Rmg8*, implying that they may be homologous, at least from a breeding perspective (Anh et al., 2018). *Rmg7* and *Rmg8* showed resistance at both the seedling and head stages (Tagle et al., 2015; Anh et al., 2018). The resistance of *Rmg7* is reported to be overcome by recent MoT strains (Cruz and Valent, 2017). *RmgGR119* was identified in the Albanian wheat landrace GR119 and was found to be effective against many MoT isolates. This landrace also has *Rmg8*, indicating that *Rmg8* and *RmgGR119* collectively conferred a good level of blast resistance (Wang et al., 2018). However, their performance in field conditions needs to be tested before being utilized in a breeding program. Both *Rmg7* and *Rmg8* work fine at 21–24°C; however, as the temperature goes over 26°C, *Rmg7* loses its resistance, whereas *Rmg8* remains effective (Anh et al., 2018).

Apart from the *Rmg* genes, some *R* genes with broad spectrum resistance might also confer WB resistance. *Lr34* is a non-NBS-LRR gene belonging to the ABC transporter gene family, exhibiting durable resistance against rusts, powdery mildew, and spot blotch. Krattinger et al. (2016) demonstrated its effectiveness against rice blast in a transgenic Nipponbare variety, implying its possible resistance to WB. Accumulation of minor genes by eliminating highly resistant and susceptible individuals in advanced segregating populations has been tried in rice blast resistance breeding (Khush and Jena, 2009) and could be used in WB resistance breeding as well.

The above genes were identified in greenhouse experiments conducted mostly at the seedling stage. In field experiments, however, resistance to WB appears to be more of quantitative resistance. An example in this regard was reported by He et al. (2020b), in which the 2NS translocation explained 22.4–50.1% of the blast variation across diverse environments in the Caninde#1/Alondra mapping population. Additional minor quantitative trait loci (QTL) were identified on chromosomes 1AS, 2BL, 3AL, 4BS, 4DL, and 7BS, acting in an additive mode to 2NS translocation. In another study, Goddard et al. (2020) mapped WB resistance in two mapping populations and identified five QTL for seedling blast resistance on chromosomes 2B, 4B, 5A, and 6A, and four QTL for head blast resistance on chromosomes 1A, 2B, 4A, and 5A, and concluded that the common resistant parent BR 18-Terena had quantitative resistance against WB. Additionally, genome-wide association studies (GWASs) for field WB resistance have been reported in international nurseries of CIMMYT (Juliana et al., 2019, 2020) and in a diverse panel of lines from South Asia and CIMMYT (He et al., 2021). The common finding was that the 2NS translocation was the only major and consistent resistance locus, whereas loci on other chromosome regions were of low phenotypic effects and were not stably expressed across experiments.

Juliana et al. (2019) performed a GWAS on a panel of 271 wheat-breeding lines from CIMMYT that was evaluated for field response to wheat blast in Quirusillas, Bolivia. They reported the association of *Qcim.2A.1* in the position of the 2NS translocation, and a locus on chromosome 3BL with field blast resistance. In another study, Juliana et al. (2020) performed a large multi-environment GWAS using 8,607 observations on 1,106 lines from CIMMYT, to identify genomic regions associated with field blast resistance in Bolivia and Bangladesh. They identified 36 markers on chromosomes 2AS, 3BL, 4AL, and 7BL that were consistently associated with blast resistance in different environments, with more than half of them tagging the 2NS translocation and explaining up to 71.8% of the blast variation. A recent GWAS on field and greenhouse resistance to wheat blast was done by He et al. (2021) using a diverse panel of 184 genotypes from South Asia and CIMMYT. While the authors identified a significant marker trait associations on chromosomes 1BS, 2AS, 6BS, and 7BL, only those on chromosome 2AS were consistent in the different datasets.

### Genomic Selection

Given the critical need to shift focus from breeding for qualitative blast resistance to quantitative resistance, genomic selection (GS) is a promising tool that can accelerate genetic gains, reduce cycle time, and facilitate accurate selection for quantitative disease resistance (Poland and Rutkoski, 2016). In GS, a training “population” comprising individuals with whole-genome marker data and phenotypes is used to train prediction models and estimate marker effects, which are then used to obtain genomic-estimated breeding values of individuals that have not been phenotyped but only genotyped (referred to as “selection candidates”) (Meuwissen et al., 2001). As several studies have demonstrated GS to be promising for rice blast (Huang et al., 2019) and wheat diseases such FHB, rusts, Septoria tritici blotch, Stagonospora nodorum blotch, and tan spot (Rutkoski et al., 2012; Juliana et al., 2017a, b), it is an attractive breeding strategy that can be effectively integrated in wheat blast resistance breeding to minimize time, cost, and resources for blast phenotyping in the field. In addition, GS can be potentially used by breeding programs to select individuals for resistant line advancement and crossing prior to phenotyping, and to increase the selection intensity by scaling-up selections for blast resistance to early generations of the breeding cycle, where large segregating populations pose a challenge for blast evaluation.

### Mutation Breeding Potential

The AVR gene product (effectors) of the blast pathogen interacts with “R” genes to confer resistance to the disease. The “R” genes and plant defense machinery are under constant selection pressure due to pathogen evolution and hence newer “R” genes are evolving by spontaneous mutation events such as natural recombination, gene duplication, and uneven crossing over. However, the low frequency of spontaneous mutation viz. 1 in 10^6^ per gene necessitates the need for induced mutagenesis (Kozjak and Meglic, 2012). Mutation breeding has evolved from the use of physical and chemical mutagens to genomics technologies of modern times such as RNA interference (RNAi) using siRNA and miRNA, virus-induced gene silencing (VIGS), *Agrobacterium*-mediated insertional mutagenesis (AIM), and targeting-induced local lesions in genome (TILLING), all of which have a potential in breeding for WB resistance. The advantage with the modern techniques includes precise site-directed mutagenesis in genes of interest. Physical mutagens (α and β rays, X-rays, γ-irradiation, etc.) cause high amount of DNA damage/rearrangements as compared with chemical mutagens (EMS, MMS, sodium azide, etc.) and, thus, the latter are preferred for creating point mutation (e.g., EMS used in TILLING population), which may provide gain or loss of gene function (Kozjak and Meglic, 2012). The gain or loss in function is important, in particular for targeting “R” genes, which can be modified to be recognized by multiple AVR effectors or multiple allelic forms of an AVR gene, thus, providing a broad-spectrum resistance. The “R” genes corresponding to the effectors (AVR) essential for pathogen survival are a good candidate for durable resistance (Vleeshouwers and Oliver, 2014). The number of currently known “R” genes for WB is low. Hence, mutations can help in identifying novel “R” genes along with modifying the existing ones for improving WB resistance.

According to the IAEA database, mutation breeding programs in different countries lead to the release of 69 wheat cultivars resistant to various fungal infections. Wheat variety Dharkhan-172 developed using sodium azide as mutagen is latest in the series that was released in Mongolia in 2018. It was resistant against spot blotch, loose smut, and stripe rust and moderately resistant to Septoria nodorum blotch (SNB) and Alternaria leaf blight^1^. Recently in Bangladesh, gamma radiation has been used in wheat seeds for obtaining mutant resistant lines against WB, and some of the mutated plants exhibited improved WB resistance (Rashid et al., 2019). Mutation breeding has also been successful in developing Ug99-resistant wheat varieties in Kenya. More than 34 M_1_ populations and around 284,000 M_1_ plants were grown and screened under the field and greenhouse conditions and the material after M_4_ generation narrowed down to four resistant entries, out of which two cultivars, namely, Eldo Ngano1 and Eldo Mavuno1, were released for the farmers of Kenya in 2014 (Bado, 2015). Thus, type of the mutagen to be used, population size to handle in subsequent generations, identification of the mutant and their preservation from the natural out-crossing (especially recessive mutations) are some of the important factors to be considered while breeding for resistance to diseases, such as WB, which requires more research initiatives in the upcoming times.

### Biotechnology That Includes Gene Editing

Biotechnology has proven to be an effective tool in modern breeding for most of the important crop plant species, especially in areas where conventional breeding has reached its limits. Sequence information (wheat and pathogen), bioinformatics tools, and DNA based markers have much contributed toward crop improvement including breeding for disease resistance. DNA markers, especially SNPs, are being used to locate QTLs for resistance to diseases such as WB. Studies on effective QTLs conferring field blast resistance in wheat are very few, and the available ones have not identified major and stable QTLs beyond the 2AS/2NS translocation (He et al., 2020b; Juliana et al., 2020). Sequence information is also utilized to differentiate strains based on differential DNA fingerprinting. SSR marker (Pereira et al., 2014), transposons *viz.* Pot2 (Kachroo et al., 1994), MGR586 (Farman et al., 1996), and *grh* retroelement (Dobinson et al., 1993), etc., can be used for detection and classification of MoT isolates. Technologies such as conventional PCR, quantitative real-time PCR (qPCR), LAMP, recombinase polymerase amplification (RPA), and nucleic acid lateral flow immunoassay (NALFIA) are used for pathogen detection (Kang et al., 2020; Thierry et al., 2020), for which MoT-specific markers as determinant factors are still being developed and validated as discussed before.

In recent years, CRISPR/Cas9 has been demonstrated to be a powerful tool for the improvement of crops *via* genome editing. It can be done either by stacking of “R” genes or deletion or disruption of S genes or transcription factors in the genome of commercial varieties (Wang et al., 2016; Kim et al., 2018). Genome editing has been applied to improve important crop plants, such as rice, wheat, maize, and soybean (Wang et al., 2016; Bhowmik et al., 2018). With the advent of novel genome editing tools, it is possible to create modified resistance genes through targeted gene mutagenesis such as CRISPR-Cas9 (Haque et al., 2018). A relevant example is the disruption *via* CRISPR-Cas9 of a blast susceptibility gene in rice *OsERF922*, which enhanced the resistance to rice blast (Wang et al., 2016). In wheat, CRISPR-Cas9 has been used to disrupt various genes such as *TaDREB2* and *TaERF3* (Kim et al., 2018), demonstrating its strong potentiality in mutating WB susceptibility genes once identified. The availability of wheat genomic resources and the molecular biology of regulation of blast resistance response in rice might help in the identification of target genes for genome editing in wheat for MoT resistance.

## Agronomic Management

Management of wheat blast calls for the adoption of integrated disease management approaches as its causal pathogen MoT has a wide host range including crop species and weedy grasses (Pagani et al., 2014). A live example is the extensively grown pasture grass in Brazil, i.e., *Urochloa brizantha* (signal grass), which was later found to harbor strains of MoT and may have an important role in WB epidemic (Castroagudín et al., 2017). Thus, the management of grassy hosts around wheat fields is very important, as it can reduce inoculum buildup (Mehta, 2014). Inoculum is reported to survive on crop residues and, hence, deep plowing and destruction or removal of residues is an effective strategy (Ceresini et al., 2019). However, such management protocols are not followed in WB-affected South American countries either because of its high cost or because of the prevalent conservation agricultural practices that are popular among farmers (Duveiller et al., 2016a). The highest yield reduction happens at the heading stage by airborne conidial infection coming from within the field or from the nearby secondary hosts. Nevertheless, seed treatment with fungicide is reported to limit the initial infection and inoculum buildup in the field, thereby being beneficial to WB control (Prabhu et al., 1992; Urashima et al., 1993). Rotating the cropping pattern with non-host crops, such as pulses and oilseed, can help in minimizing inoculum density and reduce disease pressure (Pagani et al., 2014). However, it is difficult to apply this strategy in practice because of the wide range of alternative hosts of MoT that significantly limit the crops in rotation with wheat. Indeed, studies on the effects of rotation with prevalent crops (maize, soybean, mucuna, crotalaria) in South America were performed, but the results were not encouraging (Kohli et al., 2020). Another important issue with farmers in affected South American countries is their tendency to use a high seeding rate. The idea was to get more spikes to compensate for the loss of some tillers due to the disease, but this practice may lead to earliness in flowering and dense canopy micro-climate conducive for WB development, which ultimately may increase yield loss (Kohli et al., 2020).

Adjustment in planting date is another effective mitigating strategy against the disease. Congenial conditions for the disease include warmer temperature (25–30°C), long wet hours of the spike (25–40 h), and high relative humidity (>90%); thus, planting dates have to be decided considering the local conditions. Rains during the flowering stage followed by hot and humid days can lead to disease development (Kohli et al., 2011). Early planting in Brazil, Bolivia, and Paraguay (before 10–20th April) is highly risky, as the flowering coincides with ambient conditions favorable for WB development (Kohli et al., 2020). Hence, sowing is recommended in May. However, in Bangladesh, avoiding the late sown conditions (after 30th November) was effective for managing blast, as rains and humidity coincide with heading under late sown conditions (He et al., 2020a). Kohli et al. (2020) recommended the use of a variety combination with genotypes differing in maturity and WB resistance, in the hope to reduce the amount of field inoculum. Along with timely planting, treating seed with thiram and carboxin, and prophylactic foliar spray of triazoles and strobilurins were found to be effective in managing WB in Bangladesh (Roy et al., 2020a).

## Mineral Nutrition and Additives for Managing Wheat Blast

Various elements and chemicals such as silicon (Si), magnesium (Mg), calcium (Ca), potassium phosphate, potassium silicate, and ethephon are reported to affect blast resistance by altering physiological pathways in a plant (Cruz et al., 2011). For efficient working of the photosynthetic machinery and scavenging of the reactive oxygen species (ROS), a plant needs high Si and low Mg in the nutrition (Rodrigues et al., 2017). Calcium is needed to induce defense-related genes, but high Mg reduces Ca in the plant and makes it susceptible to blast disease (Debona et al., 2016). Likewise, potassium phosphate and ethephon are reported to enhance resistance against blast (Cruz et al., 2011).

Blast infection in wheat reduces the activity of the enzyme RUBISCO, net carbon assimilation, and photosynthetic activity. This results in lowered accumulation of storage and soluble sugars, i.e., glucose, fructose, sucrose, and ultimately, reduction in storage starch in grains (Debona et al., 2016; Rios et al., 2017). The application of silicon is reported to enhance incubation period and limit disease progression. Si has been hypothesized to provide mechanical support by depositing below the cuticle in epidermal and collenchyma cells of the spike of wheat and, hence, physically limiting pathogen penetration (Cruz M.F.A. et al., 2015). It stimulates flavonoid accumulation inside the epidermal cell, which may lead to the activation of many defense genes. Expression levels of various defense-related genes that are involved in the salicylic acid (SA) and jasmonic acid (JA) pathways *viz.* pathogenesis-related1 (PR-1), β-1,3-glucanase, chitinase, peroxidizes, phenylalanine ammonialyase, etc., were significantly expressed in higher amounts when provided with external silicon (Cruz M.F.A. et al., 2015) and calcium (Debona et al., 2017). Genotypic difference in response to silicate application has been observed in WB (Pagani et al., 2014), indicating that there is a need to screen out the genotypes responding better to external application of chemicals. Another positive effect exerted by silicon is the higher expression of ROS-scavenging enzymes. Upon WB infection, ROS triggers defense genes in wheat; and, at the same time, they cause lipid peroxidation of cell membranes, resulting in loss of photosynthetic pigments and machinery (Debona et al., 2012). Hence, the scavenging of ROS becomes necessary for the plant. Silicon increases the activities of antioxidant enzymes such as superoxide dismutase (SOD), peroxidase (POX), and ascorbate peroxidases (APX), which in turn reduce ROS (Debona et al., 2012).

High doses of nitrogen have been associated with increased blast severity, where the relative growth of the fungal mycelia is enhanced, especially in varieties with high nitrogen use efficiency (Ballini et al., 2013). In fact, resistance genes are reported to be moderated by the levels of nitrogen. *Pi1* gene in rice was partially broken down with high doses of nitrogen. However, there are genes independent from the dose of nitrogen, e.g., *Pia* gene remains effective under high nitrogen (Ballini et al., 2013). Thus, identification and utilization of the latter type of genes are beneficial in managing WB in a high-nitrogen regime.

The importance of iron (Fe) against *M. oryzae* was found in rice where high Fe supply and down-regulation of a Fe transporter macrophage protein gene *OsNramp6* resulted in enhanced resistance against rice blast (Peris-Peris et al., 2017). Therefore, wheat NRAMP6 homologs might play a similar role in resistance to WB, which needs to be validated in later studies.

## Disease Modeling and Forecasting

There is a need for WB forecasting, so that prophylactic control measures can be taken well in advance for minimizing losses due to the disease. Several researchers have tried to develop models based on humidity and temperature, the two most important factors for WB development (Alves and Fernandes, 2006). The optimal temperature of 30°C with no less than 10 h wetting period may result in WB development. However, with wetting hours exceeding 40, the disease may develop even at 25°C (Cardoso et al., 2008). The 2009 epidemic in Parana coincided with the heavy rainfall received during June and July, again emphasizing the importance of high humidity (Duveiller et al., 2016a). Remote sensing can be utilized to identify spectral signatures for WB. Healthy and blast-infected plants can be differentiated by spectral signatures between 650 and 1050 nm wavelength, using a handheld spectro-radiometer in farmer fields in Bangladesh (Yesmin et al., 2020), which can be scaled up *via* mounting multispectral cameras on drones, aeroplanes, or even satellites. Fernandes et al. (2017) developed a model based on weather parameters, with which they correctly predicted the epidemic (2015) and non-epidemic (2016) years in Northern Paraná, Brazil. The advantage of such forecasting tools is in the ability of the tools to concern farmers and policymakers well in advance for initiating control measures such as fungicide sprays. Similar models can be made and adjusted to disease-prone areas in South Asia.

## Fungicides for Wheat Blast Management

Fungicides are currently indispensable for WB management, considering the limited effects of varietal resistance. Fungicide efficacy can be judged by its outcome on a susceptible variety, but the results are not very promising and was found to be cultivar dependent in South America while it was found effective in Bangladesh (Kohli et al., 2011; Roy et al., 2020a). Fungicides become ineffective under high-disease pressure or congenial environmental conditions against the disease (Kohli et al., 2020). It is reported that sometimes even four sprays were not able to completely control WB infection in some areas of Brazil (Urashima et al., 2017), thereby affirming the fact that genetic resistance in combination with fungicides is much needed (Ceresini et al., 2018). Although fungicides are mostly used at the heading stage, their application at the seedling stage is also important in reducing inoculum load on basal or older leaves (Cruz et al., 2015).

Both seed treatment and foliar spray with fungicides in isolation or combination have been tried against wheat blast. Infected seeds while germinating can perpetuate the growth of fungi to cotyledons and primary leaves (Buerstmayr et al., 2017). Thus, seed treatment with fungicides, such as benomyl (Sadat and Choi, 2017), difenoconazole (Yesmin et al., 2020), and carboxin + thiram (BWMRI, 2020), is recommended. A spray of mancozeb-based fungicides and a mix of QoI + DMI (quinone outside inhibitor, QoI, and demethylation inhibitors, DMI) were found effective in Brazil and Bolivia, respectively (Cruz et al., 2019). The combination of triazole and strobilurin fungicides (e.g., Nativo75 WG, Amister Top 325 SC) is also advised to farmers in Bangladesh (Sadat and Choi, 2017; BWMRI, 2020; Roy et al., 2020a). MoT isolates collected from farmer fields in Bangladesh revealed that carbendazim (Autostin 50WGD, Knowin 50WP) and QoI + DMI fungicides, *viz.* Nativo 75WG (tebuconazole + trifloxistrobin), of as low as 50 ppm were able to completely inhibit MoT mycelial growth under *in vitro* conditions. However, the two mancozeb-based fungicides used in the same study were not effective (Debnath et al., 2019).

DMI + QoI fungicides are working well in Bolivia but not in Brazil, implying different prevailing MoT isolates and influence from different climatic conditions in the two countries. Strobilurin fungicides, belonging to the QoI type that attacks mitochondrial respiration in the pathogen, were in extensive use against the disease in Brazil. In recent years, new MoT isolates with mutated mitochondrial *cytb* gene emerged, which are resistant to QoI fungicides (Castroagudin et al., 2015). During the span of 7 years (2005–2012), the frequency of this mutation has increased from 36 to 90% in the sampled population (Castroagudin et al., 2015). In recent years, a new generation of fungicide, SDHI (succinate dehydrogenase inhibitors), has been used frequently. However, it is very likely that MoT will develop resistance to SDHI fungicides if they are used singly, and hence it is recommended to use them in combination or in rotation with other types of fungicides (Ceresini et al., 2019).

## Induced Resistance Against Wheat Blast

Disease resistance can be induced in a plant *via* external stimuli, such as pathogen attack and external application of phytohormones or their inducers. The effectiveness of SA against *Magnaporthe* spp. has been reported in rice (Manandhar et al., 1998) and wheat (Rios et al., 2014). For rice blast, both foliar spray and soil drenching (but not the seed treatment) of SA limited blast infection on foliage, and the latter suggests the induced resistance to be systemic (Manandhar et al., 1998). SA activates many pathogenesis-related (PR) genes *viz.* peroxidases (POX), polyphenoloxidase (PPO), chitinase (CHI), and β-1,3-glucanase (GLU), which have been associated with WB resistance (Rios et al., 2014). In a study on two wheat cultivars, BRS-229 and BR-18, in Brazil, all three phytohormones: SA, jasmonic acid (JA) and ethylene (ET) were found effective to reduce WB, although the effectiveness of JA and ET was much higher than that of ASM (SA analog) (Rios et al., 2014). Apart from phytohormones, beneficial microorganisms can also induce resistance in the plant by various induced systemic resistance (ISR) elicitor molecules such as lipopeptides, siderophores, antibiotics, and volatile organic compounds (De Vleesschauwer et al., 2008).

Beneficial microorganisms against rice blast have been reported, in which control agents, such as bacterial strains of *Pseudomonas* spp., *Bacillus* spp. (Gnanamanickam and Mew, 1992), and *Streptomyces* spp. (Law et al., 2017), and fungi such as *Trichoderma harzianum* (Singh et al., 2012), were effective against rice blast and, therefore, hold promise against WB. Bacteria, in particular *Bacillus* spp., were reported to act against MoT either by inducing systemic resistance in wheat or releasing antagonizing antimicrobial compounds (Gilroy et al., 2017). *Pseudomonas fluorescens* strain WCS417r and *P. aeruginosa* strain 7NSK2 limited rice blast pathogen by activating JA- and ET-regulated genes (De Vleesschauwer and Hofte, 2006). *B. methylotrophicus* was able to inhibit *M. oryzae* mycelium growth in *in vitro* studies (Nascimento et al., 2016).

The containment of MoT under *in vitro* conditions has been reported in recent studies. *Streptomyces* spp. with the help of elicitor molecules, *viz.* oligomycins B and F, was able to inhibit the mycelial growth of MoT (Chakraborty et al., 2020). Lipopeptides are another class of elicitor molecule extracted from bacteria, especially *Bacillus* spp., and are reported to inhibit the growth of conidia, germ tube, and appressorium in MoO (Liao et al., 2016). Unlike fungicides, they are environment friendly, which is attributed to easy biodegradation and less toxicity. They have an additional advantage due to their receptor unspecificity, which does not assert selection pressure on *Magnaporthe* strains. Their usefulness for WB was reported from marine *B. subtilis* strain 109GGC020, from which five different extracted lipopeptides (gageotetrin B, gageopeptide C, gageopeptide D, gageopeptide A, and gageopeptide B) had an inhibitory effect on the growth of MoT either by blocking spore germination or interfering with the germ tube or appressoria formation (Chakraborty et al., 2020). There are some fungal toxins that can mimic the disease and induce resistance in plants if used in lower concentrations. Alpha-picolinic acid is a tryptophan derivative fungal toxin whose spray in lower concentration is found to lower MoT infection. It protects the photosynthetic machinery because of increased antioxidant accumulation (Aucique-Pérez et al., 2019). However, it is important to note that many of these experiments were performed under *in vitro* conditions; and, hence, the efficacy of these biocontrol agents need to be tested under field conditions before application in practice.

## Conclusion

This review updates about the spread of WB in different continents of the globe and discussed potential management approaches to mitigate this problem. Currently, wheat blast is considered as an explosive and significantly damaging disease of wheat worldwide. From its origin in Brazil in 1985, it has spread to many South American countries and then made intercontinental jumps to Bangladesh in South Asia and Zambia in Africa. Although most wheat-growing regions/countries of the world are still free from this disease, it has a potential to spread in other countries of the world especially Europe, the United States, Australia, China, India, etc., which is an alarming situation for future food security. Several management strategies for mitigating the effects of wheat blast exits, but a holistic and sustainable approach is needed. The MoT pathogen is fast-evolving, highly aggressive, and potentially devastating in various agro-ecological zones; therefore, a globally intensive effort is needed to prevent its damage and limit its introduction and spread.

## Author Contributions

All authors contributed to the information gathering, write-up and discussion of the manuscript.

## Conflict of Interest

The authors declare that the research was conducted in the absence of any commercial or financial relationships that could be construed as a potential conflict of interest.

## Publisher’s Note

All claims expressed in this article are solely those of the authors and do not necessarily represent those of their affiliated organizations, or those of the publisher, the editors and the reviewers. Any product that may be evaluated in this article, or claim that may be made by its manufacturer, is not guaranteed or endorsed by the publisher.

## Footnotes

1 https://mvd.iaea.org/#!Search

## References

[B1] Alves K. J. P. Fernandes J. M. C. (2006). Influência da temperatura e da umidade relativa do ar na esporulação de *Magnaporthe grisea* em trigo. *Fitopatol. Bras.* 31 579–584. 10.1590/S0100-41582006000600007

[B2] Anh V. L. Anh N. T. Tagle A. G. Vy T. T. P. Inoue Y. Takumi S. (2015). *Rmg8*, a new gene for resistance to *Triticum* isolates of *Pyricularia oryzae* in hexaploid wheat. *Phytopathology* 105 1568–1572. 26555672 10.1094/PHYTO-02-15-0034-R

[B3] Anh V. L. Inoue Y. Asuke S. Vy T. T. P. Anh N. T. Wang S. (2018). Rmg8 and *Rmg7*, wheat genes for resistance to the wheat blast fungus, recognize the same avirulence gene *AVR-Rmg8*. *Mol. Plant Pathol.* 19 1252–1256. 10.1111/mpp.12609 28846191 PMC6638012

[B4] Aucique-Pérez C. E. Resende R. S. Cruz N. L. B. Dornelas F. DaMatta F. M. Rodrigues F. Á (2019). Picolinic acid spray stimulates the antioxidative metabolism and minimizes impairments on photosynthesis on wheat leaves infected by *Pyricularia oryzae*. *Physiol. Plant.* 167 628–644. 10.1111/ppl.12917 30628091

[B5] Bado S. (2015). *Advances in Plant Mutation Breeding.* Ph.D. Thesis. Vienna: University of Natural Resources and Life Sciences.

[B6] Ballini E. Nguyen T. T. T. Morel J. B. (2013). Diversity and genetics of nitrogen-induced susceptibility to the blast fungus in rice and wheat. *Rice* 6 1–13. 10.1186/1939-8433-6-32 24280346 PMC4883689

[B7] Barea G. Toledo J. (1996). Identificación y Zonificación de Pyricularia o brusone (*Pyricularia oryzae*) en el cutivo de trigo en el Departamento de Santa Cruz, Informe Tecnico, Proyecto de Investigacion Trigo. Santa Cruz de la Sierra: Centro de Investigación Agrícola Tropical, 76–86.

[B8] Bhowmik P. Ellison E. Polley B. Bollina V. Kulkarni M. Ghanbarnia K. (2018). Targeted mutagenesis in wheat microspores using CRISPR/Cas9. *Sci. Rep.* 8 1–10. 29695804 10.1038/s41598-018-24690-8 PMC5916876

[B9] Buerstmayr H. Mohler V. Kohli M. (2017). “Advances in control of wheat diseases: Fusarium head blight, wheat blast and powdery mildew,” in *Achieving Sustainable Cultivation of Wheat*, ed. Langridge P. (Cambridge: Burleigh Dodds Science Publishing), 345–370. 10.19103/as.2016.0004.21

[B10] BWMRI (2020). *Annual Research Report 2019-20.* Dinajpur: Bangladesh Wheat and Maize Research Institute.

[B11] Callaway E. (2016). Devastating wheat fungus appears in Asia for first time. *Nature* 532 421–422. 10.1038/532421a 27121815

[B12] Cao X. Chen L. Zhou Y. Duan X. (2011). Potential distribution of *Magnaporthe grisea* in China and the world, predicted by MaxEnt. *Plant Prot.* 37 80–83.

[B13] Cardoso C. A. D. A. Reis E. M. Moreira E. N. (2008). Development of a warning system for wheat blast caused by *Pyricularia grisea*. *Summa Phytopathol.* 34 216–221. 10.1590/S0100-54052008000300002

[B14] Castroagudin V. L. Ceresini P. C. de Oliveira S. C. Reges J. T. A. Maciel J. L. N. Bonato A. L. V. (2015). Resistance to QoI fungicides is widespread in Brazilian populations of the wheat blast pathogen *Magnaporthe oryzae*. *Phytopathology* 105 284–294. 10.1094/phyto-06-14-0184-r 25226525

[B15] Castroagudín V. L. Danelli A. Moreira S. I. Reges J. T. A. Carvalho G. Maciel A. L. (2017). The wheat blast pathogen *Pyricularia graminis-tritici* has complex origins and a disease cycle spanning multiple grass hosts. *bioRxiv* [Preprint]. 10.1101/203455

[B16] Ceresini P. C. Castroagudín V. L. Rodrigues F. Á Rios J. A. Aucique-Pérez C. E. Moreira S. I. (2019). Wheat blast: from its origins in South America to its emergence as a global threat. *Mol. Plant Pathol.* 20 155–172. 10.1111/mpp.12747 30187616 PMC6637873

[B17] Ceresini P. C. Castroagudín V. L. Rodrigues F. Á Rios J. A. Eduardo Aucique-Pérez C. Moreira S. I. (2018). Wheat blast: past, present, and future. *Annu. Rev. Phytopathol.* 56 427–456. 10.1146/annurev-phyto-080417-050036 29975608

[B18] Chakraborty M. Mahmud N. U. Gupta D. R. Tareq F. S. Shin H. J. Islam T. (2020). Inhibitory effects of linear lipopeptides from a marine *Bacillus subtilis* on the wheat blast fungus *Magnaporthe oryzae Triticum*. *Front. Microbiol.* 11:665. 10.3389/fmicb.2020.00665 32425899 PMC7203576

[B19] Choi J. Park S. Y. Kim B. R. Roh J. H. Oh I. S. Han S.-S. (2013). Comparative analysis of pathogenicity and phylogenetic relationship in *Magnaporthe grisea* species complex. *PLoS One* 8:e57196. 10.1371/journal.pone.0057196 23468934 PMC3582606

[B20] Chuma I. Zhan S.-W. Asano S. Nga N.T.T. Vy T.T.P. Shirai M. (2010). PWT1, an avirulence gene of Magnaporthe oryzae tightly linked to the rDNA locus, is recognized by two staple crops, common wheat and barley. *Phytopathology* 100 436–443. 20373964 10.1094/PHYTO-100-5-0436

[B21] CIMMYT (2016). *Understanding and Managing the Threat of Wheat Blast in South Asia, South America, and Beyond.* Mexico: CIMMYT publication repository.

[B22] Couch B. C. Kohn L. M. (2002). A multilocus gene genealogy concordant with host preference indicates segregation of a new species, *Magnaporthe oryzae*, from *M. grisea*. *Mycologia* 94 683–693. 10.2307/3761719 21156541

[B23] Cruppe G. Cruz C. D. Peterson G. Pedley K. Asif M. Fritz A. (2020). Novel sources of wheat head blast resistance in modern breeding lines and wheat wild relatives. *Plant Dis.* 104 35–43. 10.1094/PDIS-05-19-0985-RE 31660799

[B24] Cruz C. D. Bockus W. W. Stack J. P. Tang X. Valent B. Pedley K. F. (2012). Preliminary assessment of resistance among US wheat cultivars to the *Triticum* pathotype of *Magnaporthe oryzae*. *Plant Dis.* 96 1501–1505. 10.1094/pdis-11-11-0944-re 30727304

[B25] Cruz C. D. Kiyuna J. Bockus W. W. Todd T. C. Stack J. P. Valent B. (2015). *Magnaporthe oryzae* conidia on basal wheat leaves as a potential source of wheat blast inoculum. *Plant Pathol.* 64 1491–1498. 10.1111/ppa.12414

[B26] Cruz M. F. A. Silva L. A. F. Rios J. A. Debona D. Rodrigues F. Á (2015). Microscopic aspects of the colonization of *Pyricularia oryzae* on the rachis of wheat plants supplied with silicon. *Bragantia* 74 207–214. 10.1590/1678-4499.0023

[B27] Cruz C. D. Peterson G. L. Bockus W. W. Kankanala P. Dubcovsky J. Jordan K. (2016). The 2NS translocation from *Aegilops ventricosa* confers resistance to the *Triticum pathotype* of *Magnaporthe oryzae*. *Crop Sci.* 56 990–1000. 10.2135/cropsci2015.07.0410 27814405 PMC5087972

[B28] Cruz C. D. Santana F. M. Todd T. C. Maciel J. L. N. Kiyuna J. Cruz A. P. (2019). Multi-environment assessment of fungicide performance for managing wheat head blast (WHB) in Brazil and Bolivia. *Trop. Plant Pathol.* 44 183–191. 10.1007/s40858-018-0262-9

[B29] Cruz C. D. Valent B. (2017). Wheat blast disease: danger on the move. *Trop. Plant Pathol.* 42 210–222. 10.1007/s40858-017-0159-z

[B30] Cruz M. F. A. Diniz A. P. C. Rodrigues F. A. Barros E. G. (2011). Foliar application of products on the reduction of blast severity on wheat. *Trop. Plant Pathol.* 36 424–428.

[B31] Cumagun C. J. R. Anh V. L. Vy T. T. P. Inoue Y. Asano H. Hyon G.-S. (2014). Identification of a hidden resistance gene in tetraploid wheat using laboratory strains of *Pyricularia oryzae* produced by backcrossing. *Phytopathology* 104 634–640. 10.1094/PHYTO-04-13-0106-R 24824421

[B32] Cunfer B. M. Yorinori T. Igarashi S. (1993). “Wheat blast,” in *Seed Borne Diseases and Seed Health Testing of Wheat. Danish Government Institute of Seed Pathology for Developing Countries*, eds Mathur S. B. Cunfer B. M. (Denmark: Copenhagen), 125–128.

[B33] De Vleesschauwer D. Djavaheri M. Bakker P. A. H. M. Höfte M. (2008). *Pseudomonas* fluorescens WCS374r-induced systemic resistance in rice against *Magnaporthe oryzae* is based on pseudobactin-mediated priming for a salicylic acid-repressible multifaceted defense response. *Plant Physiol.* 148 1996–2012. 10.1104/pp.108.127878 18945932 PMC2593667

[B34] De Vleesschauwer D. Hofte P. C. M. (2006). Redox-active pyocyanin secreted by *Pseudomonas aeruginosa* 7NSK2 triggers systemic resistance to *Magnaporthe grisea* but enhances *Rhizoctonia solani* susceptibility in rice. *Mol. Plant Microbe Int.* 19 1406–1419. 10.1094/MPMI-19-1406 17153925

[B35] Dean R. Van Kan J. A. Pretorius Z. A. Hammond-Kosack K. E. Di Pietro A. Spanu P. D. (2012). The top 10 fungal pathogens in molecular plant pathology. *Mol. Plant Pathol.* 13 414–430. 10.1111/j.1364-3703.2011.00783.x 22471698 PMC6638784

[B36] Debnath B. Khan A. A. Hossain M. M. Rubayet M. T. Miah M. R. U. (2019). Morphological, pathological and cultural characteristics of *Magnaporthe oryzae triticum* causing blast of wheat and its fungicidal control. *Can. J. Agr.Crop* 4 218–227. 10.20448/803.4.2.218.227

[B37] Debona D. Cruz M. F. A. Rodrigues F. A. (2017). Calcium-triggered accumulation of defense-related transcripts enhances wheat resistance to leaf blast. *Trop. Plant Pathol.* 42 309–314. 10.1007/s40858-017-0144-6

[B38] Debona D. Rios J. A. Nascimento K. J. T. Silva L. C. Rodrigues F. A. (2016). Influence of magnesium on physiological responses of wheat infected by *Pyricularia oryzae*. *Plant Pathol.* 65 114–123. 10.1111/ppa.12390

[B39] Debona D. Rodrigues F. Á Rios J. A. Nascimento K. J. T. (2012). Biochemical changes in the leaves of wheat plants infected by *Pyricularia oryzae*. *Phytopathology* 102 1121–1129. 10.1094/phyto-06-12-0125-r 22913412

[B40] Dobinson K. F. Harris R. E. Hamer J. E. (1993). Grasshopper, a long terminal repeat (LTR) retroelement in the phytopathogenic fungus *Magnaporthe grisea*. *Mol. Plant Microbe Int.* 6 114–126. 10.1094/mpmi-6-114 7679935

[B41] Dos Anjos J. R. N. Da Silva D. B. Charchar M. J. D. Rodrigues G. C. (1996). Occurrence of blast fungus (*Pyricularia grisea*) on wheat and rye in the savanna region of central Brazil. *Pesqui. Agropecu. Bras.* 3 79–82.

[B42] Duveiller E. Hodson D. Sonder K. Tiedermann A. (2011). An international perspective on wheat blast. *Phytopathology* 101 S220.

[B43] Duveiller E. He X. Singh P. K. (2016a). “Wheat blast: an emerging disease in South America potentially threatening wheat production,” in *World Wheat Book: A History of Wheat*, Vol. 3 eds Bonjean A. van Ginkel M. (Paris: Lavoisier), 1107–1122.

[B44] Duveiller E. Hodson D. von Tiedemann A. Islam S. Ahmed Z. Sonder K. (2016b). “Modeling and mapping wheat disease risk,” in *Proceedings of the Oral Presentation Regional Consultation Workshop on Mitigating the Threat of Wheat Blast in Bangladesh and Beyond*, Kathmandu.

[B45] Farman M. L. (2002). *Pyricularia grisea* isolates causing gray leaf spot on perennial ryegrass (*Lolium perenne*) in the United States: relationship to *P. grisea* isolates from other host plants. *Phytopathology* 92 245–254. 10.1094/phyto.2002.92.3.245 18943995

[B46] Farman M. L. Taura S. Leong S. A. (1996). *Magnaporthe grisea* DNA fingerprinting probe MGR586 contains the 3’ end of an inverted repeat transposon. *Mol. Gen. Genet.* 251 675–681. 10.1007/bf02174116 8757398

[B47] Fernandes J. M. C. Nicolau M. Pavan W. Hölbig C. A. Karrei M. Karrei de Vargas F. (2017). A weather-based model for predicting early season inoculum build-up and spike infection by the wheat blast pathogen. *Trop. Plant Pathol.* 42 230–237. 10.1007/s40858-017-0164-2

[B48] Ferreira J. R. Torres G. A. M. Consoli L. Binneck E. Camilotti G. A. Scagliusi S. (2020). “Genetic and molecular basis of wheat-*Magnaporthe oryzae Triticum*interaction,” in *Wheat Blast*, eds Kumar S. Kashyap P. L. Singh G. P. (Boca Raton, FL: CRC Press), 69–104. 10.1201/9780429470554-5

[B49] Gilroy E. M. Taylor R. M. Hein I. Boevink P. Sadanandom A. Joe W. (2017). Genomic analyses reveal that biocontrol of wheat blast by *Bacillus spp*. may be linked with production of antimicrobial compounds and induced systemic resistance in host plants. *Mol. Biol. Evol.* 1:201710683. 10.6084/m9.figshare.5852661.v1

[B50] Gnanamanickam S. S. Mew T. W. (1992). Biological control of blast disease of rice (*Oryza sativa* L.) with antagonistic bacteria and its mediation by a *Pseudomonas* antibiotic. *Jpn. J. Phytopathol.* 58 380–385. 10.3186/jjphytopath.58.380

[B51] Goddard R. Steed A. Chinoy C. Ferreira J. R. Scheeren P. L. Maciel J. N. L. (2020). Dissecting the genetic basis of wheat blast resistance in the Brazilian wheat cultivar BR 18-Terena. *BMC Plant Biol.* 20:398. 10.1186/s12870-020-02592-0 32854622 PMC7451118

[B52] Goulart A. C. P. Paiva F. A. (1992). Incidence of (*Pyricularia oryzae*) in different wheat cultivars under field conditions. *Fitopatol. Bras*. 17 321–325.

[B53] Goulart A. C. P. Paiva F. A. Mesquita A. N. (1990). Occurrence of wheat blast (*Pyricularia oryzae*) in the state of Mato Grosso do Sul. *Fitopatol. Bras.* 15 112–114.

[B54] Goulart A. C. P. Sousa P. G. Urashima A. S. (2007). Damages in wheat caused by infection of *Pyricularia grisea*. *Summa Phytopathol*. 33 358–363.

[B55] Gutiérrez S. A. Cúndom M. A. (2015). *Pyricularia oryzae* en cultivos de cebada en Corrientes (Argentina). *Summa Phytopathol.* 41 318–320. 10.1590/0100-5405/2063

[B56] Haque E. Taniguchi H. Hassan M. M. Bhowmik P. Karim M. R. Śmiech M. (2018). Application of CRISPR/Cas9 genome editing technology for the improvement of crops cultivated in tropical climates: Recent progress, prospects, and challenges. *Front. Plant Sci.* 9:617. 10.3389/fpls.2018.00617 29868073 PMC5952327

[B57] He X. Gupta V. Bainsla N. K. Chawade A. Singh P. K. (2020a). “Breeding for wheat blast resistance,” in *Wheat Blast*, eds Kumar S. Kashyap P. L. Singh G. P. (Boca Raton, FL: CRC Press), 163–174. 10.1201/9780429470554-9

[B58] He X. Kabir M. R. Roy K. K. Anwar M. B. Xu K. Marza F. (2020b). QTL mapping for field resistance to wheat blast in the Caninde#1/Alondra population. *Theor. Appl. Genet.* 133 2673–2683. 10.1007/s00122-020-03624-x 32488302 PMC7419448

[B59] He X. Juliana P. Kabir M. R. Roy K. K. Islam R. Marza F. (2021). Screening and mapping for head blast resistance in a panel of CIMMYT and South Asian bread wheat germplasm. *Front Genet.* 12:679162. 10.3389/fgene.2021.679162 34054928 PMC8155635

[B60] Helguera M. Khan I. A. Kolmer J. Lijavetzky D. Zhong-qi L. Dubcovsky J. (2003). PCR assays for the *Lr37-Yr17-Sr38* cluster of rust resistance genes and their use to develop isogenic hard red spring wheat lines. *Crop Sci.* 43 1839–1847. 10.2135/cropsci2003.1839

[B61] Hossain A. Mottaleb K. A. Farhad M. Barma N. C. (2019). Mitigating the twin problems of malnutrition and wheat blast by one wheat variety, “BARI Gom 33”, in Bangladesh. *Acta Agrobot.* 72:1775. 10.5586/aa.1775

[B62] Huang M. Balimponya E. G. Mgonja E. M. Emmanuel M. McHale L. Leah K. (2019). Use of genomic selection in breeding rice (*Oryza sativa* L.) for resistance to rice blast (*Magnaporthe oryzae*). *Mol. Breed.* 39:114. 10.1007/s11032-019-1023-2

[B63] ICAR-IIWBR (2020). *Progress Report of AICRP on Wheat and Barley 2019-20, Crop Protection.* Karnal: ICAR-Indian Institute of Wheat and Barley Research.

[B64] Igarashi S. (1990). “Update on wheat blast (Pyricularia oryzae) in Brazil,” in *Proceedings of International Conference Wheat Nontraditional Warm Areas*, ed. Saunders D. (Mexico: CIMMYT), 480–483.

[B65] Igarashi S. Utiamada C. M. Igarashi L. C. Kazuma A. H. Lopes R. S. (1986). *Pyricularia* in wheat.1. Occurrence of *Pyricularia* sp. in Paraná State. *Fitopatol. Bras.* 11 351–352.

[B66] Inoue Y. Vy T. T. P. Yoshida K. Asano H. Mitsuoka C. Asuke S. (2017). Evolution of the wheat blast fungus through functional losses in a host specificity determinant. *Science* 357 80–83. 10.1126/science.aam9654 28684523

[B67] Islam M. T. Croll D. Gladieux P. Soanes D. M. Persoons A. Bhattacharjee P. (2016). Emergence of wheat blast in Bangladesh was caused by a South American lineage of *Magnaporthe oryzae*. *BMC Biol.* 14:84. 10.1186/s12915-016-0309-7 27716181 PMC5047043

[B68] Juliana P. He X. Kabir M. R. Roy K. K. Anwar M. B. Marza F. (2020). Genome-wide association mapping for wheat blast resistance in CIMMYT’s international screening nurseries evaluated in Bolivia and Bangladesh. *Sci. Rep.* 10 1–14. 10.1038/s41598-020-72735-8 33009436 PMC7532450

[B69] Juliana P. Poland J. Huerta-Espino J. Shrestha S. Crossa J. Crespo-Herrera L. (2019). Improving grain yield, stress resilience and quality of bread wheat using large-scale genomics. *Nat. Genet.* 51 1530–1539. 10.1038/s41588-019-0496-6 31548720

[B70] Juliana P. Singh R. P. Singh P. K. Crossa J. Huerta-Espino J. Lan C. (2017a). Genomic and pedigree-based prediction for leaf, stem, and stripe rust resistance in wheat. *Theor. Appl. Genet.* 130 1415–1430. 10.1007/s00122-017-2897-1 28393303 PMC5487692

[B71] Juliana P. Singh R. P. Singh P. K. Crossa J. Rutkoski J. E. Poland J. A. (2017b). Comparison of models and whole-genome profiling approaches for genomic-enabled prediction of Septoria tritici blotch, Stagonospora nodorum blotch, and tan spot resistance in wheat. *Plant Genome* 10:82. 10.3835/plantgenome2016.08.0082 28724084

[B72] Kachroo P. Leong S. A. Chattoo B. B. (1994). Pot2, an inverted repeat transposon from the rice blast fungus *Magnaporthe grisea*. *Mol. Gen. Genet.* 245 339–348. 10.1007/bf00290114 7816044

[B73] Kang H. Peng Y. Hua K. Deng Y. Bellizzi M. Gupta D. R. (2020). Rapid detection of wheat blast pathogen *Magnaporthe oryzae triticum* pathotype using genome-specific primers and Cas12a-mediated technology. *Engineering.* 10.1016/j.eng.2020.07.016

[B74] Kang S. Chumley F. G. Valent B. (1994). Isolation of the mating-type genes of the phytopathogenic fungus *Magnaporthe grisea* using genomic subtraction. *Gene* 138 289–296. 10.1093/genetics/138.2.289 PMC1206148 7828813

[B75] Kato H. Yamamoto M. Yamaguchi-Ozaki T. Kadouchi H. Iwamoto Y. Kadouchi H. (2000). Pathogenicity, mating ability and DNA restriction fragment length polymorphisms of *Pyricularia* populations isolated from Gramineae, Bambusideae and Zingiberaceae plants. *J. Gen. Plant Pathol.* 66 30–47. 10.1007/pl00012919

[B76] Khush G. S. Jena K. K. (2009). Advances in genetics, genomics and control of rice blast disease. *Adv. Genet. Genom. Control Rice Blast Dis.* 54 1–10. 10.1007/978-1-4020-9500-9

[B77] Kim D. Alptekin B. Budak H. (2018). CRISPR/Cas9 genome editing in wheat. *Funct. Integr. Genom.* 18 31–41. 10.1007/s10142-017-0572-x 28918562

[B78] Kohli M. M. Cazal C. Chavez A. (2020). “Integrated management of wheat blast disease,” in *Wheat Blast*, eds Kumar S. Kashyap P. L. Singh G. P. (Boca Raton, FL: CRC Press), 175–190. 10.1201/9780429470554-10

[B79] Kohli M. M. Mehta Y. R. Guzman E. de Viedma L. Cubilla L. E. (2011). Pyricularia blast-a threat to wheat cultivation. *Czech. J. Genet. Plant Breed.* 47 130–134. 10.17221/3267-cjgpb

[B80] Kozjak P. Meglic V. (2012). “Mutagenesis in plant breeding for disease and pest resistance,” in *Mutagenesis*, ed. Mishra R. (London: IntechOpen), 195–220. 10.5772/50332

[B81] Krattinger S. G. Sucher J. Selter L. L. Chauhan H. Zhou B. Tang M. (2016). The wheat durable, multipathogen resistance gene *Lr34* confers partial blast resistance in rice. *Plant Biotechnol. J.* 14 1261–1268. 10.1111/pbi.12491 26471973 PMC11388880

[B82] Law J. W. F. Ser H. L. Khan T. M. Chuah L. H. Pusparajah P. Chan K. C. (2017). The potential of streptomyces as biocontrol agents against the rice blast fungus, *Magnaporthe oryzae* (*Pyricularia oryzae*). *Front. Microbiol.* 8:3. 10.3389/fmicb.2017.00003 28144236 PMC5239798

[B83] Liao J. H. Chen P. Y. Yang Y. L. Kan S. C. Hsieh F. C. Liu Y. C. (2016). Clarification of the antagonistic effect of the lipopeptides produced by *Bacillus amyloliquefaciens* BPD1 against *Pyricularia oryzae* via in situ MALDI-TOF IMS analysis. *Molecules* 21:1670. 10.3390/molecules21121670 27918491 PMC6273258

[B84] Maciel J. L. N. (2019). “Diseases affecting wheat: wheat blast,” in *Integrated Disease Management in Wheat and Barley*, ed. Oliver R. (London: Burleigh Dodds Science Publication), 155–169. 10.19103/as.2018.0039.08

[B85] Maciel J. L. N. Ceresini P. C. Castroagudin V. L. Zala M. Kema G. H. J. McDonald B. A. (2014). Population structure and pathotype diversity of the wheat blast pathogen *Magnaporthe oryzae* 25 years after its emergence in Brazil. *Phytopathology* 104 95–107. 10.1094/phyto-11-12-0294-r 23901831

[B86] Malaker P. K. Barma N. C. D. Tiwari T. P. Collis W. J. Duveiller E. Singh P. K. (2016). First report of wheat blast caused by *Magnaporthe oryzae* pathotype *triticum* in Bangladesh. *Plant Dis.* 100:2330.

[B87] Manandhar H. K. Lyngs Jørgensen H. J. Mathur S. B. Smedegaard-Petersen V. (1998). Resistance to rice blast induced by ferric chloride, di-potassium hydrogen phosphate and salicylic acid. *Crop Prot.* 17 323–329. 10.1016/S0261-2194(98)00020-9

[B88] Mehta Y. R. (2014). “Pillars of integrated disease management,” in *Wheat Diseases and their Management*, ed. Mehta Y. R. (New York, NY: Springer International Publishing), 17–63. 10.1007/978-3-319-06465-9_2

[B89] Meuwissen T. H. E. Hayes B. J. Goddard M. E. (2001). Prediction of total genetic value using genome-wide dense marker maps. *Genetics* 157 1819–1829. 10.1093/genetics/157.4.1819 11290733 PMC1461589

[B90] Moreira S. I. Ceresini P. C. Alves E. (2015). Reprodução sexuada em *Pyricularia oryzae*. *Summa Phytopathol.* 41 175–182. 10.1590/0100-5405/2067

[B91] Mottaleb K. A. Singh P. K. He X. Hossain A. Kruseman G. Erenstein O. (2019). Alternative use of wheat land to implement a potential wheat holiday as wheat blast control: In search of feasible crops in Bangladesh. *Land Use Policy* 82 1–12. 10.1016/j.landusepol.2018.11.046

[B92] Mottaleb K. A. Singh P. K. Sonder K. Kruseman G. Tiwari T. P. Barma N. C. D. (2018). Threat of wheat blast to South Asia’s food security: an ex-ante analysis. *PLoS One* 13:e0197555. 10.1371/journal.pone.0197555 29782528 PMC5962063

[B93] Murakami J. Tosa Y. Kataoka T. Tomita R. Kawasaki J. Chuma I. (2000). Analysis of host species specificity of *Magnaporthe grisea*toward wheat using a genetic cross between isolates from wheat and foxtail millet. *Phytopathology* 90 1060–1067. 10.1094/phyto.2000.90.10.1060 18944467

[B94] Nascimento I. O. Rodrigues A. A. C. Moraes F. H. de Sousa F. A. Corsi M. C. F. Catarino A. M. (2016). Isolation, identification and in vitro evaluation of *Bacillus spp.* in control of *Magnaporthe oryzae* comparing evaluation methods. *Afr. J. Agric. Res.* 11 1743–1749. 10.5897/AJAR2016.10931

[B95] Nga N. T. T. Hau V. T. B. Tosa Y. (2009). Identification of genes for resistance to a Digitaria isolate of *Magnaporthe grisea* in common wheat cultivars. *Genome* 52 801–809. 10.1139/g09-054 19935928

[B96] Pagani A. P. S. Dianese A. C. Café-Filho A. C. (2014). Management of wheat blast with synthetic fungicides, partial resistance and silicate and phosphite minerals. *Phytoparasitica* 42 609–617. 10.1007/s12600-014-0401-x

[B97] Patkar R. N. Benke P. I. Qu Z. Chen Y. Y. Yang F. Swarup S. (2015). A fungal monooxygenase-derived jasmonate attenuates host innate immunity. *Nat. Chem. Biol.* 11 733–740. 10.1038/nchembio.1885 26258762

[B98] Pereira J. F. Consoli L. De Souza Bombonatto E. A. Bonato A. L. V. Maciel J. L. N. (2014). Development of genomic SSR markers and molecular characterization of *Magnaporthe oryzae* isolates from wheat in Brazil. *Biochem. Genet.* 52 52–70. 10.1007/s10528-013-9627-4 24271825

[B99] Perelló A. Martinez I. Molina M. (2015). First report of virulence and effects of *Magnaporthe oryzae* isolates causing wheat blast in Argentina. *Plant Dis.* 99:1177. 10.1094/pdis-11-14-1182-pdn

[B100] Perelló A. E. Martinez I. Sanabria A. Altamirano R. Sibole J. V. (2017). Pathogenicity of isolates of *Magnaporthe spp.* from wheat and grasses infecting seedlings and mature wheat plants in Argentina. *Plant Pathol.* 7 1149–1161. 10.1111/ppa.12658

[B101] Peris-Peris C. Serra-Cardona A. Sánchez-Sanuy F. Campo S. Ariño J. San Segundo B. (2017). Two NRAMP6 isoforms function as iron and manganese transporters and contribute to disease resistance in rice. *Mol. Plant Microbe Int.* 30 385–398. 10.1094/MPMI-01-17-0005-R 28430017

[B102] Picinini E. C. Fernandes J. M. C. (1990). Occurrence of wheat blast *Pyricularía oryzae* in commercial fields in the State of Rio Grande do Sul Brazil. *Fitopatol. Bras.* 15 83–84.

[B103] Pieck M. L. Ruck A. Farman M. L. Peterson G. L. Stack J. P. Valent B. (2017). Genomics-based marker discovery and diagnostics assay development for wheat blast. *Plant Dis*. 101 103–109. 10.1094/pdis-04-16-0500-re 30682315

[B104] Poland J. Rutkoski J. (2016). Advances and challenges in genomic selection for disease resistance. *Annu Rev Phytopathol* 54 79–98. 10.1146/annurev-phyto-080615-100056 27491433

[B105] Prabhu A. S. Filippi M. C. Castro N. (1992). Pathogenic variation among isolates of *Pyricularia oryzae* infecting rice, wheat, and grasses in Brazil. *Trop. Pest Manag.* 38 367–371. 10.1080/09670879209371729

[B106] Prestes A. Arendt P. Fernandes J. Scheeren P. (2007). “Resistance to *Magnaporthe grisea* among Brazilian wheat genotypes,” in *Wheat Production in Stressed Environments*, eds Buck H. T. Nisi J. E. Salomon N. (Cham: Springer), 119–123. 10.1007/1-4020-5497-1_16

[B107] Rashid M. H. Meah M. B. Uddin M. I. Ahmed S. Kashem M. A. (2019). Gamma radiated wheat for combating devastating blast disease (*Magnaporthe oryzae Triticum*) in Bangladesh. *Agril. Sci.* 1 1–10.

[B108] Reis E. Blum M. C. Forcelini C. (1995). Sobrevivência de *Pyricularia oryzae*, associada as sementes de trigo. *Summa Phytopathol.* 21 43–44. 10.17801/0101-3122/rbs.v20n1p43-47

[B109] Rios J. A. Debona D. Duarte H. S. S. Rodrigues F. A. (2013). Development and validation of a standard area diagram set to assess blast severity on wheat leaves. *Eur. J. Plant Pathol.* 136 603–611. 10.1007/s10658-013-0191-x

[B110] Rios J. A. Rios V. S. Aucique-Pérez C. E. Cruz M. F. A. Morais L. E. DaMatta F. M. (2017). Alteration of photosynthetic performance and source-sink relationships in wheat plants infected by *Pyricularia oryzae*. *Plant Pathol.* 66 1496–1507. 10.1111/ppa.12693

[B111] Rios J. A. Rodrigues F. Á Debona D. Resende R. S. Moreira W. R. Andrade C. C. L. (2014). Induction of resistance to *Pyricularia oryzae* in wheat by acibenzolar-S-methyl, ethylene and jasmonic acid. *Trop. Plant Pathol.* 39 224–233. 10.1590/S1982-56762014000300006

[B112] Rodrigues F. Á Rios J. A. Debona D. Aucique-Pérez C. E. (2017). *Pyricularia oryzae*-wheat interaction: physiological changes and disease management using mineral nutrition and fungicides. *Trop. Plant Pathol.* 42 223–229. 10.1007/s40858-017-0130-z

[B113] Roy K. K. Anwar B. Mustarin K. Reza M. M. A. Rahman M. M. E. Malaker P. K. (2020a). Evaluation of different fungicides (chemical, botanical and bio-agent) in controlling wheat blast in a blast prone area in Bangladesh. *Arch. Phytopathol.* 10.1080/03235408.2020.1827652

[B114] Roy K. K. Rahman M. M. E. Reza M. M. A. Mustarin K. E. Malaker P. K. Barma N. C. D. (2020b). First report of triticale blast caused by the fungus *Magnaporthe oryzae* pathotype *Triticum* in Bangladesh. *Can. J. Plant Pathol.* 43 288–295. 10.1080/07060661.2020.1793223

[B115] Roy K. K. Rahman M. M. E. Mustarin K. Reza M. M. A. Barma N. C. D. He X. (2021a). First report of barley blast caused by *Magnaporthe oryzae* pathotype *Triticum* in Bangladesh. *J. Gen. Plant Pathol.* 87 184–191. 10.1007/s10327-021-00985-z

[B116] Roy K. K. Rahman M. M. E. Mustarin K. Reza M. M. A. Barma N. C. D. He X. (2021b). First report of durum wheat (*Triticum turgidum* var. *durum*) blast caused by the fungus *Magnaporthe oryzae* pathotype *Triticum* in Bangladesh. *Phytopathol. Mediterranea* 60 107–113.

[B117] Rutkoski J. Benson J. Jia Y. Brown-Guedira G. Jannink J.-L. Sorrells M. (2012). Evaluation of genomic prediction methods for Fusarium head blight resistance in wheat. *Plant Genome* 5 51–61. 10.3835/plantgenome2012.02.0001

[B118] Sadat M. A. Choi J. (2017). Wheat blast: A new fungal inhabitant to Bangladesh threatening world wheat production. *Plant Pathol. J.* 33 103–108. 10.5423/ppj.rw.09.2016.0179 28381956 PMC5378430

[B119] Saharan M. S. Bhardwaj S. C. Chatrath R. Sharma P. Choudhary A. K. Gupta R. K. (2016). Wheat blast disease—an overview. *J. Wheat Res.* 8 1–5. 10.1201/9780429470554-1

[B120] Singh D. P. (2017). Wheat blast−A new challenge to wheat production in South Asia. *Indian Phytopathol*. 70 169–177.

[B121] Singh P. K. Singh A. K. Singh H. B. Dhakad B. K. (2012). Biological control of rice blast disease with *Trichoderma harzianum* in direct seeded rice under medium low land rainfed conditions. *Environ. Ecol.* 30 834–837.

[B122] Singh R. P. Singh P. K. Rutkoski J. Hodson D. P. He X. Jørgensen L. N. (2016). Disease impact on wheat yield potential and prospects of genetic control. *Annu. Rev. Phytopathol.* 54 303–322. 10.1146/annurev-phyto-080615-095835 27296137

[B123] Tagle A. G. Chuma I. Tosa Y. (2015). *Rmg7*, a new gene for resistance to *Triticum* isolates of *Pyricularia oryzae* identified in tetraploid wheat. *Phytopathology* 105 495–499. 10.1094/PHYTO-06-14-0182-R 25870924

[B124] Takabayashi N. Tosa Y. Oh H. S. Mayama S. (2002). A gene-for-gene relationship underlying the species-specific parasitism of Avena/Triticum isolates of *Magnaporthe grisea* on wheat cultivars. *Phytopathology* 92 1182–1188. 10.1094/PHYTO.2002.92.11.1182 18944243

[B125] Tembo B. Mulenga R. M. Sichilima S. M’siska K. K. Mwale M. Chikoti P. C. (2020). Detection and characterization of fungus (*Magnaporthe oryzae* pathotype *Triticum*) causing wheat blast disease on rain-fed grown wheat (*Triticum aestivum* L.) in Zambia. *PLoS One* 15:e0238724. 10.1371/journal.pone.0238724 32956369 PMC7505438

[B126] Thierry M. Chatet A. Fournier E. Tharreau D. (2020). A PCR, qPCR, and LAMP toolkit for the detection of the wheat blast pathogen in seeds. *Plants* 9:277. 10.3390/plants9020277 32098075 PMC7076445

[B127] Thierry M. Gladieux P. Fournier E. Tharreau D. Ioos R. (2019). A genomic approach to develop a new qPCR test enabling detection of the *Pyricularia oryzae* lineage causing wheat blast. *Plant Dis.* 104 60–70. 10.1094/PDIS-04-19-0685-RE 31647693

[B128] Tosa Y. Chuma I. (2014). Classification and parasitic specialization of blast fungi. *J. Gen. Plant Pathol.* 80 202–209. 10.1007/s10327-014-0513-7

[B129] Tosa Y. Hirata K. Tamba H. Nakagawa S. Chuma I. Isobe C. (2004). Genetic constitution and pathogenicity of Lolium isolates of *Magnaporthe oryzae* in comparison with host species-specific pathotypes of the blast fungus. *Phytopathology* 94 454–462. 10.1094/phyto.2004.94.5.454 18943763

[B130] Tosa Y. Tamba H. Tanaka K. Mayama S. (2006). Genetic analysis of host species specificity of *Magnaporthe oryzae* isolates from rice and wheat. *Phytopathology* 96 480–484. 10.1094/PHYTO-96-0480 18944307

[B131] Tufan H. A. McGrann G. R. Magusin A. Morel J. B. Miché L. Boyd L. A. (2009). Wheat blast: histopathology and transcriptome reprogramming in response to adapted and non-adapted *Magnaporthe* isolates. *New Phytol*. 184 473–484. 10.1111/j.1469-8137.2009.02970.x 19645735

[B132] Urashima A. S. Alves A. F. Silva F. N. Oliveira D. Gazaffi R. (2017). Host range, mating type and population structure of *Magnaporthe sp.* of a single barley field in São Paulo state. *Brazil J. Phytopathol.* 165 414–424. 10.1111/jph.12575

[B133] Urashima A. S. Galbieri R. Stabili A. (2005). DNA fingerprinting and sexual characterization revealed two distinct populations of *Magnaporthe grisea* in wheat blast from Brazil. *Czech. J. Genet. Plant Breed.* 41 238–245. 10.17221/6184-cjgpb

[B134] Urashima A. S. Gross C. R. F. Stabili A. Freitas E. G. Silva C. P. Netto D. C. S. (2009). “Effect of *Magnaporthe grisea* on seed germination, yield and quality of wheat,” in *Advances in Genetics, Genomics and Control of Rice Blast Disease*, eds Xiaofan W. Valent B. (New York, NY: Springer), 267–277. 10.1007/978-1-4020-9500-9_27

[B135] Urashima A. S. Hashimoto Y. Don Y. Kusaba M. Tosa Y. Nakayashiki H. (1999). Molecular analysis of the wheat blast population in Brazil with a homolog of retrotransposon MGR583. *Ann. Phytopathol. Soc. Jpn.* 65 429–436. 10.3186/jjphytopath.65.429

[B136] Urashima A. S. Igarashi S. Kato H. (1993). Host range, mating type, and fertility of *Pyricularia grisea* from wheat in Brazil. *Plant Dis.* 77 1211–1216. 10.1094/pd-77-1211

[B137] Urashima A. S. Kato H. (1994). Varietal resistance and chemical control of wheat blast fungus. *Summa Phytopathol.* 20 107–112.

[B138] Urashima A. S. Leite S. F. Galbieri R. (2007). Efficiency of aerial dissemination of *Pyricularia grisea*. *Summa Phytopathol.* 33 275–279.

[B139] Valent B. Cruz C. D. Farman M. Peterson G. L. Pedley K. (2016). “Strategies for managing blast disease of wheat,” in *Achieving Durable Resistance to Wheat Diseases and Pests*, eds Hao W. Gao Y. (Minneapolis, MI: American Phytopathology Society), 5.

[B140] Vales M. Anzoátegui T. Huallpa B. Cazon M. I. (2018). Review on resistance to wheat blast disease (*Magnaporthe oryzaeTriticum*) from the breeder point-of-view: use of the experience on resistance to rice blast disease. *Euphytica* 214:1. 10.1007/s10681-017-2087-x

[B141] Viedma L. (2005). Wheat blast occurrence in Paraguay. *Phytopathology* 95:152.

[B142] Viedma L. Q. Kohli M. Cubilla L. Cabrera G. (2010). “Manejo integrado de mancha amarilla y la *Piricularia* en el cultivo de trigo en Paraguay,” in *Tercer Seminario Nacional Detrigo del Grano al Pan*, eds Kohli M. Cubilla L. E. Cabrera G. (Asunción: Parag), 31–42.

[B143] Viedma L. Q. Morel W. (2002). *A~nublo o Piricularia del Trigo (WheatBlast).* D’ıptico: Parag.

[B144] Vleeshouwers V. G. A. A. Oliver R. P. (2014). Effectors as tools in disease resistance breeding against biotrophic, hemibiotrophic, and necrotrophic plant pathogens. *Mol. Plant Microbe Int.* 27 196–206. 10.1094/MPMI-10-13-0313-IA 24405032

[B145] Vy T. T. P. Hyon G. Nga N. T. T. Inoue Y. Chuma I. Tosa Y. (2014). Genetic analysis of host-pathogen incompatibility between Lolium isolates of *Pyricularia oryzae* and wheat. *J. Gen. Plant Pathol.* 80 59–65. 10.1007/s10327-013-0478-y

[B146] Wang F. J. Wang C. L. Liu P. Q. Lei C. L. Hao W. Gao Y. (2016). Enhanced rice blast re- sistance by CRISPR/Cas9-targeted mutagenesis of the ERF transcription factor gene *OsERF922*. *PLoS One* 11:e0154027. 10.1371/journal.pone.0154027 27116122 PMC4846023

[B147] Wang S. Asuke S. Vy T. T. P. Inoue Y. Chuma I. Win J. (2018). A new resistance gene in combination with *Rmg8* confers strong resistance against triticum isolates of *Pyricularia oryzae* in a common wheat landrace. *Phytopathology* 108 1299–1306. 10.1094/PHYTO-12-17-0400-R 29767554

[B148] Wise K. Woloshuk C. (2010). *Diseases of Wheat :Fusarium Head Blight (Head Scab).* West Lafayette: Purdue University.

[B149] Yan X. Talbot N. J. (2016). Investigating the cell biology of plant infection by the rice blast fungus *Magnaporthe oryzae*. *Curr. Opin. Microbiol.* 34 147–153. 10.1016/j.mib.2016.10.001 27816794

[B150] Yasuhara-Bell J. Pedley K. F. Farman M. Valent B. Stack J. P. (2018). Specific detection of the wheat blast pathogen (*Magnaporthe oryzae Triticum*) by loop-mediated isothermal amplification. *Plant Dis.* 102 2550–2559. 10.1094/pdis-03-18-0512-re 30320534

[B151] Yesmin N. Jenny F. Abdullah H. M. Hossain M. M. Kader M. A. Solomon P. S. (2020). A review on South Asian wheat blast: the present status and future perspective. *Plant Pathol.* 69 1618–1629. 10.1111/ppa.13250

[B152] Zhan S. W. Mayama S. Tosa Y. (2008). Identification of two genes for resistance to *Triticum* isolates of *Magnaporthe oryzae* in wheat. *Genome* 51 216–221. 10.1139/G07-094 18356957

[B153] Zhang H. L. Wu Z. S. Wang C. F. Li Y. Xu J. R. (2014). Germination and infectivity of microconidia in the rice blast fungus *Magnaporthe oryzae*. *Nat. Commun.* 5:4518. 10.1038/ncomms5518 PMC4143928 25082370

